# Unmet care needs among older people in residential care: a scoping review

**DOI:** 10.1186/s12877-026-07636-y

**Published:** 2026-05-12

**Authors:** Fen Xie, Louise Daly, Ludan Tang, Jessica Eustace-Cook, Anne-Marie Brady

**Affiliations:** 1 Trinity Centre for Practice and Healthcare Innovation, School of Nursing and Midwifery, Trinity College Dublin, Dublin, D02 PN40 Ireland; 2 Trinity College library, Trinity College Dublin, Dublin, D02 PN40 Ireland

**Keywords:** Unmet care needs, older people, residential care, scoping review

## Abstract

**Background:**

As the proportion of older people increases, the demand for health and social care is expected to rise substantially, along with the potential for unmet care needs. For individuals whose needs cannot be adequately met by home or community-based services due to various health and social reasons, residential care settings may provide an alternative. However, evidence indicates that the care needs of older people in residential care (RC) are often unmet or only partially met. The review aimed to map and summarise the existing literature to identify unmet care needs among older people residing in RC and explore the factors that influence the delivery of appropriate care.

**Method:**

The Joanna Briggs Institute (JBI) scoping review methodology, a priori scoping review protocol, and PRISMA-ScR guidelines were followed. A comprehensive search of seven databases (CINAHL, EMBASE, PsycINFO, MEDLINE, Web of Science, CNKI and Wan Fang) and grey literature was conducted on the 2nd of March 2024 and updated on the 30th of June 2025.

**Results:**

A total of 40 studies were included in this scoping review, most of which were conducted in Europe. Methodologically, 52.5% employed quantitative designs. The findings indicate that older adults living in RC settings experience diverse unmet care needs across the physical, psychological, social, and environmental domains, with considerable variation across regions. The review identified recurring factors at the micro, meso, and macro levels, encompassing individual characteristics, institutional practices and resource constraints, and broader systemic and policy-level factors.

**Conclusion:**

Evidence suggests that older adults living in RC settings experience diverse unmet care needs which vary across regions, and appear to be influenced by the interaction of micro, meso, and macro factors.

**Trial registration:**

This scoping review protocol was registered in the Open Science Framework on 6 December 2023 (DOI 10.17605/OSF.IO/NVRKE).

**Supplementary Information:**

The online version contains supplementary material available at 10.1186/s12877-026-07636-y.

## Background

The World Health Organization [1] reports that in 2019, there were 1 billion people aged 60 years and over worldwide, a figure projected to rise by 2.1 billion by 2050. The increasing number of people surviving to an older age is undoubtedly regarded as one of the greatest achievements of the 20th century [2], enabling more people to enjoy additional years of life. While increased longevity is a cause for celebration [3–5], older adults are more likely to experience chronic illnesses, such as stroke, Ischaemic heart disease, hypertension, chronic obstructive pulmonary disease (COPD), diabetes, and lung cancer [6–9]. Furthermore, the increasing prevalence of comorbidities in later life [10, 11] contributes to a range of physiological and psychological health challenges [12–14]. Consequently, as the proportion of older people continues to grow, so will the demand for health and social care, which will increase the potential for unmet care needs, particularly without equitable distribution and accessibility of these services [15–18].

Unmet care needs are when a person does not receive any help in resolving a specific difficulty, or the help received is considered insufficient or inappropriate [19]. Previous studies reported that unmet care needs adversely affect the quality of life and health outcomes of older people, causing a higher prevalence of severe depressive symptoms [20], reduced quality of life [21], and higher caregiver burden [22]. In addition, unmet care needs are also regarded as a key indicator of equity in the accessibility and utilisation of healthcare services [18]. Indeed, in comparison to those whose needs are met, older people with unmet needs are more likely to experience higher healthcare utilisation [23], higher risk of hospitalisations [24], increased hospital readmission rates [25], and increased risk of mortality [26]. For those persons, whose needs are not met by health and social care services at home and in the community due to various health and social reasons [27], care needs can be met in residential care (RC) facilities that are designed to provide comprehensive professional person-centred care for older people. Residential care refers to a congregate living arrangement that provides varying levels of support, including long-term care facilities, nursing homes, and assisted living settings [28, 29]. However, evidence has demonstrated that inequities in care provision persist even within RC settings, with needs sometimes unmet or only partially met [27, 30, 31]. When these needs are not met, this can lead to negative experiences, including reduced quality of life [32] and increased use of acute healthcare services [33, 34]. Therefore, effectively meeting the unmet care needs of older people is not only key to improving older adults’ quality of life [21], but also an ongoing challenge.

Although research into the care needs of older people has advanced in recent years [35–37], the specific needs and research priorities of older people in RC care settings are underexplored. The unmet care needs of older adults are multidimensional and complex. To address this complexity, this review adopts the Camberwell Assessment of Need for the Elderly (CANE) to systematically identify needs across physical, psychological, social, and environmental domains. In addition, a micro‑meso‑macro framework is used to synthesise influencing factors at the individual, organisational, and policy levels. Therefore, this scoping review aims to map and summarise the existing literature to identify unmet care needs among older people residing in RC settings and explore the factors that influence the delivery of appropriate care.

## Methods

### Design

A scoping review was deemed the most suitable review methodology, as it enables a comprehensive and systematic overview of the available evidence on a given phenomenon while providing a foundation for identifying gaps and informing potential future research [38–40]. The scoping review was conducted using the Joanna Briggs Institute (JBI) Scoping Review Methodological Framework and guided by an a priori review-specific protocol [41–43]. Additionally, the Preferred Reporting Items for Systematic Reviews and Meta-Analyses extension for scoping reviews checklist (PRISMA-ScR) was used to support the transparent and structured reporting of the scoping review [44].

Given that scoping reviews aim to provide an overview or map of the evidence, this review did not involve a quality appraisal of the included evidence sources [39, 45, 46].

### Search methods

#### Inclusion criteria

The Population, Concept and Context (PPC) framework [42, 47] was used to define the search strategy and the inclusion criteria. This framework focused the literature search, minimised bias in study selection, and established a rigorous foundation for the review.

##### Population

This scoping review included older people aged 60 years and over living in RC (For this review, the definition of older people as those “aged 60 years and over” provided by the World Health Organisation [1] was employed.)

##### Concept

Unmet care needs were defined as situations in which a person either did not receive any assistance to address a specific difficulty, or received help that was insufficient or inappropriate [19].

##### **Context**

All types of RC settings were included: residential care, long term care facility, nursing home, and assisted living facility, to comprehensively capture the unmet care needs of older adults in different institutional care settings. Geographical restrictions were not imposed.

##### Types of evidence sources

This scoping review included all types of study designs published in peer-reviewed journals and relevant grey literature examined the unmet care needs of older adults living in RC settings, such as theses, policies, and guidelines. Commentaries, letters, editorials, case reports, narratives, protocols, posters, or conference abstracts were excluded as these sources were unlikely to contain relevant information to answer the review aim.

#### Search strategy

The search strategy aimed to locate published studies that explored the unmet care needs of older people living in residential care. Given the language expertise of the primary reviewer, studies published in English or Chinese were included. The following seven databases, CINAHL Ultimate (EBSCO), EMBASE (Elsevier), PsycINFO (EBSCO), MEDLINE (EBSCO), Web of Science (Clarivate), CNKI, and Wan Fang were systematically searched for articles published from inception to 2nd of March 2024 and updated on the 30th of June 2025. Grey literature sources included in the search strategy included ProQuest Dissertations & Theses Global. Additionally, key organizational and governmental websites were searched to identify relevant policies and guidelines relevant in the subject area. These included: the World Health Organization (WHO), the United Nations Department of Economic and Social Affairs (UNDESA), the United Nations Population Fund (UNFPA), and the Organization for Economic Co-operation and Development (OECD). However, due to insufficient detail on the inclusion criteria in this review, policies and guidelines were excluded. Finally, manual searches of the reference lists of the included studies were undertaken to identify any additional citations relevant to the scoping review.

The search strategy for this scoping review was developed with the assistance of a subject specialist librarian (JEC). The search strategy utilised a combination of index terms and keywords derived from three core concepts: older people, unmet care needs, and residential care. For each concept search, a search string was developed combining index terms and keywords. The Boolean operators were applied hierarchically to combine search terms: “OR” integrated synonyms and controlled vocabulary terms within each conceptual group, while “AND” linked different conceptual groups to narrow the search scope. Database-specific functions (wildcards, quotation marks, and proximity searches) were also incorporated to optimize retrieval precision. The search string is available in *Additional file 1*.

#### Study selection

All identified citations were collated and uploaded into the reference management software EndNote X9 (Clarivate Analytics, PA, United States), and duplicates were removed. Citation details of potentially relevant papers were then imported into Covidence software (Veritas Health Innovation, Melbourne, Australia, Available at www.covidence.org), which was used to facilitate the process of title, abstract, and full text review of all remaining search results.

After the removal of duplicates, two independent reviewers (FX and LDT) screened and evaluated the citation abstracts and titles and full text according to the scoping review inclusion criteria. Any disagreements were resolved through discussion and consensus between the two reviewers; unresolved disagreements subsequently involved to resolve by a third reviewer (AMB or LD).

#### Data extraction

Data were extracted from included studies by one independent reviewer (FX) via a standardised and piloted data extraction form using the template from the JBI guidelines [41]. Two additional reviewers (AMB and LD) independently cross-checked 10% of the study characteristics for the included studies. Following this, the data extraction form was adjusted, and the remaining studies were extracted by one independent reviewer (FX). Data extraction included study characteristics such as author (s), year of publication, country, aims, study design, data collection method, sample size, data analysis, and main findings.

#### Data analysis

Data were analysed using descriptive statistical methods and basic content analysis as recommended by the JBI scoping review guideline [42, 43]. The results of the scoping review were summarised and presented using frequencies, tables, and visual figures. The Camberwell Assessment of Need for the Elderly (CANE) [48] was employed to classify the unmet care needs identified in the scoping review. The CANE was selected because it is a comprehensive assessment tool for older adults that captures physical, psychological, social, and environmental dimensions, enabling a systematic identification of their multifaceted needs. Item classification was completed by an independent researcher (FX). Studies employing alternative assessment tools or qualitative methods were mapped to the most appropriate CANE items based on their content. In addition, the “micro-meso-macro” framework [49] was employed to synthesise the issues that impact the delivery of appropriate care to older people. Originally rooted in economics, this framework is now widely used in healthcare research and to analyse how individual, institutional, and policy factors interact to produce complexity and inequality in health and healthcare services [50–53]. In this review, the “micro-meso-macro” level is defined as outlined in Table 1.

**Table 1:** The “micro-meso-macro” framework as defined in this review

Framework	Defined
Micro-level (the level of the individual residents)	Refers to the individual physiological, psychological, and social attributes of older people living in RC settings, including physical status, emotional, and social needs.
Meso-level (the level of organizations)	Refers to organizational factors such as the operation management and resource allocation of RC.
Macro-level (the policy/ /health system/contextual level)	Refers to systemic factors such as the policy system, health system and social and cultural environment of a country or region.

## Results

### Selection of sources of evidence

**Fig. 1:**
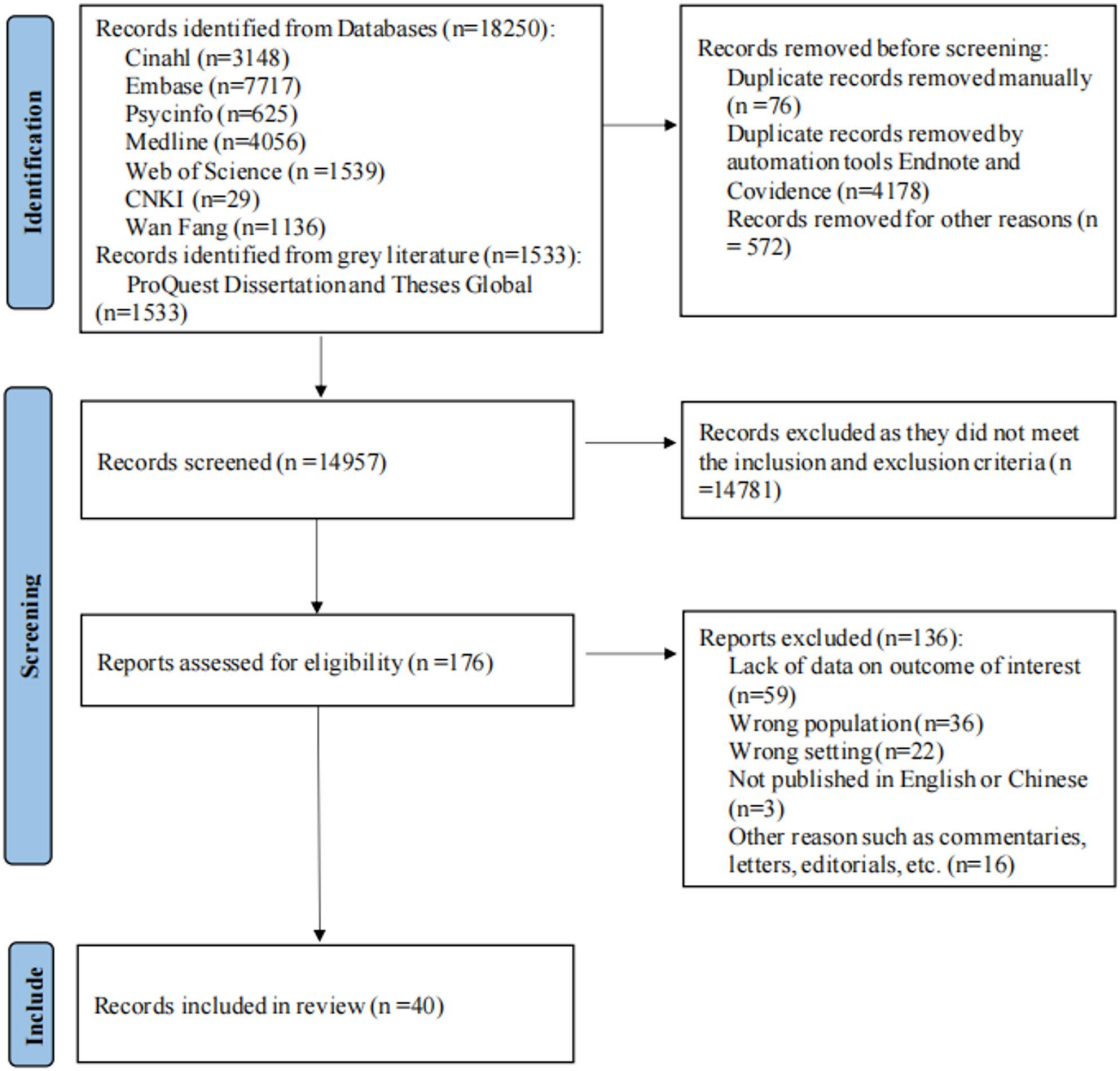
PRISMA flowchart for study selection adapted from Tricco et al. (2018) [44]

A total of 18,250 citations were identified through the database searches, and an additional 1533 citations were retrieved from grey literature sources. Following the removal of duplicates, the titles and abstracts of the remaining 14,957 citations were screened according to the inclusion criteria. Of these, 14,781 citations were excluded. A total of 176 citations were deemed potentially relevant and retrieved for full-text review. After full text assessment, 136 studies were excluded for the following reasons: lack of data on the outcome of interest (*n* = 59), wrong population (*n* = 36), wrong setting (*n* = 22), not published in English or Chinese (*n* = 3), and other reasons such as commentaries, letters, editorials, etc. (*n* = 16). The final review included 40 studies (8 published in Chinese and 32 in the English language). The study selection process is illustrated in Fig. 1.

### Characteristics of included studies

The studies included in this review reflect a broad geographical distribution, with the majority conducted in Europe (*n* = 17) followed by Asia (*n* = 14), North America (*n* = 9) (see Table 2). Most studies employed cross-sectional designs (*n* = 32, accounting for 80.0%), with surveys being the most common (*n* = 17). Further details are presented in *Additional file 2*.

**Table 2:** Summary of the characteristics of the included studies (*n* = 40)

Characteristic	*N*	%
Country		
Europe	**17**	**42.5**
Poland	6	15.0
United Kingdom	5	12.5
Portugal	2	5.0
France	1	2.5
Germany	1	2.5
Netherlands	1	2.5
Sweden	1	2.5
North America	**9**	**22.5**
United States	8	20.0
Canada	1	2.5
Asia	**14**	**35.0**
China	12	30.0
Lebanon	1	2.5
Malaysia	1	2.5
Methodology		
Cross-sectional survey	32	80.0
Cohort	3	7.5
Phenomenology	1	2.5
RCT	3	7.5
Quasi-experimental	1	2.5

**Fig. 2:**
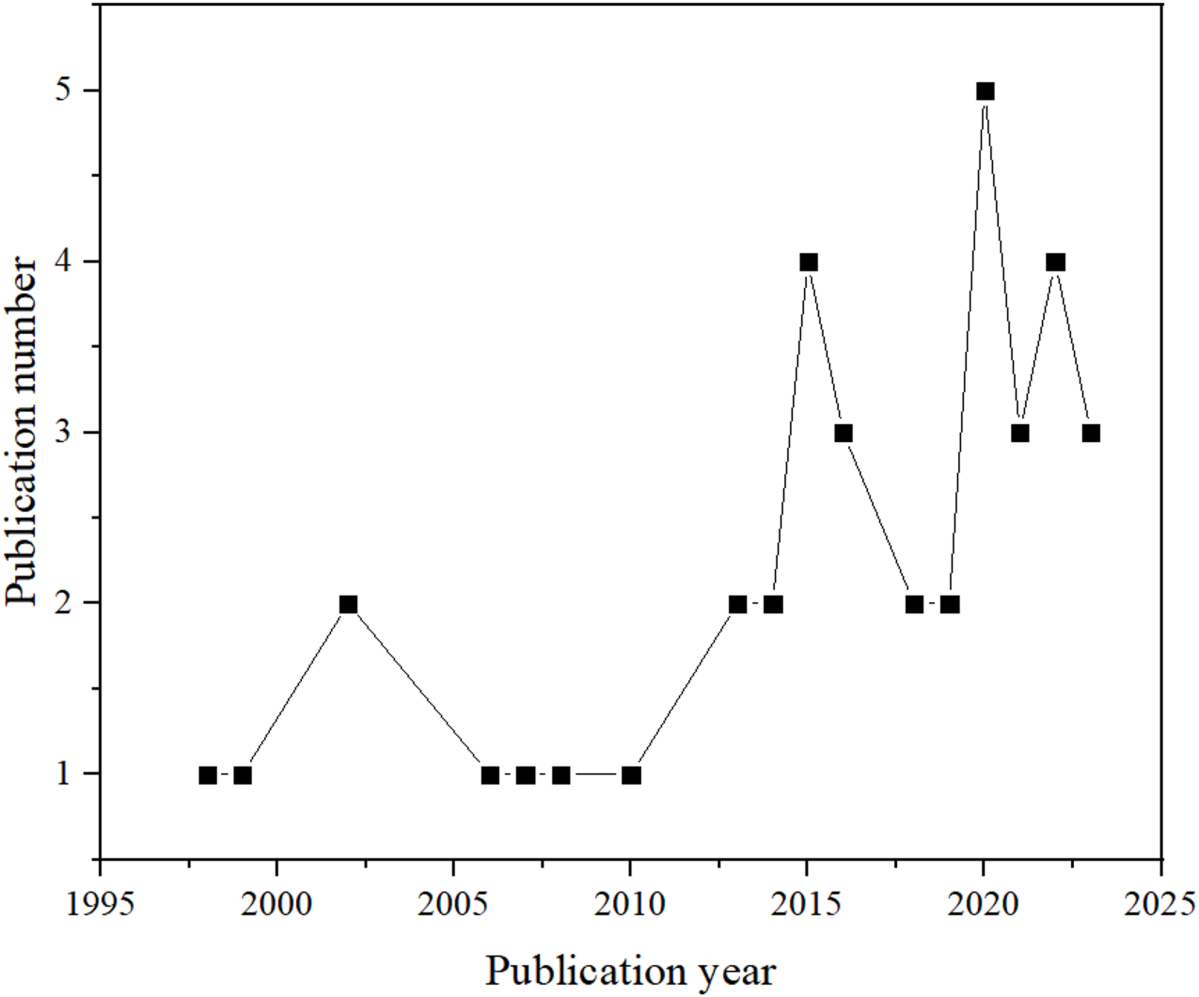
Trend of publication years (*n* = 40)

The years of publication ranged from 1998 to 2024. From 1998 to 2010, the publication activity remained static with only one publication during that period. However, since 2013, there has been a moderate increase in the research output, rising from two publications in 2013 to five in 2020, and three in 2024. These publication trends are shown in Fig. 2.

### Unmet care needs

This review summarises the frequency of unmet care needs categories reported across studies. Among the 40 included studies, a total of 308 unmet need instances were identified across four main domains: physical (*n* = 92, 29.87%), psychological (*n* = 79, 25.65%), social (*n* = 88, 28.57%), and environmental (*n* = 49, 15.91%).

Within the physical domain, the most reported unmet care needs across studies included physical health (*n* = 19, 20.65%), self-care (*n* = 18, 19.57%), eyesight/hearing/communication (*n* = 17, 18.48%), and mobility (*n* = 17, 18.48%). In the psychological domain, psychological distress (*n* = 22, 27.85%) and memory issues (*n* = 14, 17.72%) were frequently identified as unmet care needs. Social unmet needs were most often reported in relation to daytime activities (*n* = 25, 28.41%), companionship (*n* = 23, 26.14%), and intimate relationships (*n* = 18, 20.45%).

Geographical analysis revealed notable regional differences in the frequency distribution of reported categories of unmet care needs. Among the 17 studies conducted in Europe, unmet care need categories were across multiple domains, reflected regional variations in care priorities, service provision, and contextual factors, with frequently reported categories including eyesight/hearing/communication, psychological distress, memory issues, intimate relationships, companionship, and daytime activities (each *n* = 12). In North America (*n* = 9 studies), unmet care needs were reported across a narrower range of categories, suggesting differences in study focus and contextual emphasis, including mobility (*n* = 4), self-care (*n* = 3), psychological distress (*n* = 3), and daytime activities (*n* = 3). In Asia (*n* = 14 studies), studies commonly reported companionship (*n* = 8), daytime activities (*n* = 8), intimacy (*n* = 6), and psychological distress (*n* = 6). See Table 3 and 4.

**Table 3:** Overview of unmet care needs categories

Categories	Study	Martin et al. (2002) [54]	Hancock et al. (2006) [27]	Orrell et al. (2007) [55]	Orrell et al. (2008) [56]	van der Ploeg et al. (2013) [31]	Roszmann et al. (2014) [57]	Pretty (2014) [58]	Mazurek et al. (2015) [59]	Szczepańska-Gieracha et al. (2015) [60]	Wieczorowska-Tobis et al. (2016) [61]	Ferreira et al. (2016) [62]	Tobis et al. (2018) [63]	Ferreira et al. (2020) [64]	Tobis et al. (2021) [65]	Chammem et al. (2021) [66]	Schweighart et al. (2022) [67]	Liljegren et al. (2024) [68]
	Country	Europe																
		UK	UK	UK	UK	Netherlands	Poland	UK	Poland	Poland	Poland	Portugal	Poland	Portugal	Poland	France	Germany	Sweden
Unmet physical care needs	Continence	✓	✓	✓	✓	✓	✓					✓	✓					
	Mobility	✓	✓	✓	✓	✓	✓		✓		✓	✓	✓					✓
	Physical health	✓	✓	✓	✓	✓	✓	✓	✓		✓	✓	✓					
	Eyesight/hearing/communication	✓	✓	✓	✓	✓	✓		✓		✓	✓	✓	✓	✓			
	Drugs		✓	✓	✓	✓						✓	✓					
	Self-care	✓	✓	✓	✓	✓	✓		✓				✓			✓	✓	✓
Unmet psychological needs	Psychological distress	✓	✓	✓	✓	✓	✓		✓		✓	✓	✓	✓	✓			
	Psychotic symptoms	✓	✓	✓	✓	✓	✓		✓		✓	✓	✓					
	Memory	✓	✓	✓	✓	✓	✓		✓		✓	✓	✓	✓			✓	
	Behavior	✓	✓	✓	✓		✓				✓	✓	✓					
	Alcohol	✓	✓	✓							✓	✓	✓					
	Deliberate self-harm		✓	✓					✓		✓	✓	✓					
	Inadvertent self-harm	✓	✓	✓	✓	✓	✓					✓	✓					
Unmet social needs	Intimate relationships	✓	✓	✓	✓	✓	✓		✓		✓	✓	✓		✓	✓		
	Company	✓	✓	✓	✓	✓	✓		✓		✓	✓	✓	✓	✓			
	Abuse/neglect	✓	✓	✓	✓				✓			✓						
	Daytime activities	✓	✓	✓	✓	✓	✓					✓	✓	✓	✓	✓	✓	✓
	Information		✓	✓	✓	✓	✓		✓		✓	✓	✓					
Unmet environmental needs	Accommodation		✓	✓	✓	✓	✓											
	Looking after the home	✓	✓	✓	✓	✓	✓		✓									
	Food	✓	✓	✓	✓	✓	✓						✓					
	Money/budgeting		✓	✓	✓	✓	✓		✓		✓	✓	✓					
	Benefits		✓	✓	✓	✓			✓		✓	✓	✓					
	Caring for someone else	✓	✓	✓					✓				✓					
Total		18	24	24	21	19	18	1	16	0	14	19	21	5	5	3	3	3

Europe includes UK (United Kingdom), Netherlands, Poland, Portugal, France, Germany, and Sweden. North America includes United States and Canada. Asia includes China, Lebanon and Malaysia

**Table 4:** Overview of unmet care needs categories

Categories	Study	Hawkins et al. (1998) [69]	Yee et al. (1999) [70]	Kane et al. (2002) [71]	Kiely et al. (2010) [72]	Mitchell (2013) [73]	Cohen-Mansfield et al. (2015) [74]	Rivera et al. (2020) [75]	Duan et al. (2020) [76]	David et al. (2023) [77]	Nikmat and Almashoor (2015) [78]	Wang (2017) [79]	Lu (2018) [80]	Wang et al. (2019) [81]	Ma (2019) [82]	Choufani et al. (2020) [83]	Song et al. (2020) [84]	Duan (2021) [85]	Zhu (2022) [86]	Huang et al. (2022) [87]	Chen (2022) [88]	Lee et al. (2023) [89]	Huang (2023) [90]	Yuan et al. (2024) [91]
	Country	North America									Asia													
		Canada	United States	United States	United States	United States	United States	United States	United States	United States	Malaysia	China	China	China	China	Lebanon	China	China	China	China	China	China	China	China
Unmet physical care needs	Continence			✓		✓					✓									✓				
	Mobility		✓	✓		✓		✓			✓									✓				
	Physical health	✓					✓				✓	✓				✓	✓			✓				✓
	Eyesight/hearing/communication				✓						✓								✓	✓				✓
	Drugs					✓					✓									✓				
	Self-care			✓	✓	✓						✓		✓						✓			✓	
Unmet psychological needs	Psychological distress				✓		✓			✓	✓		✓	✓				✓		✓			✓	✓
	Psychotic symptoms																			✓				
	Memory										✓									✓				
	Behavior										✓									✓				
	Alcohol																							
	Deliberate self-harm																			✓				
	Inadvertent self-harm																			✓				
Unmet social needs	Intimate relationships										✓			✓				✓	✓	✓	✓			
	Company		✓					✓			✓	✓	✓	✓				✓		✓	✓		✓	✓
	Abuse/neglect										✓									✓				
	Daytime activities			✓			✓		✓		✓	✓		✓	✓			✓	✓	✓	✓			✓
	Information				✓						✓	✓								✓		✓		
Unmet environmental needs	Accommodation																							
	Looking after the home																			✓				
	Food		✓								✓									✓				
	Money/budgeting										✓													
	Benefits										✓									✓				
	Caring for someone else										✓													
Total		1	3	4	4	4	3	2	1	1	17	5	2	5	1	1	1	4	3	20	3	1	3	5

Europe includes UK (United Kingdom), Netherlands, Poland, Portugal, France, Germany, and Sweden. North America includes United States and Canada. Asia includes China, Lebanon and Malaysia

### Factors that influence the delivery of appropriate care

The findings from the included studies indicate that the provision of appropriate care to meet older adults’ needs is influenced by factors operating at three levels: Macro-level (policy/health system/contextual), Meso-level (organisational), and Micro-level (individual residents). Table 5 summarises the barriers and facilitators influencing the provision of care.

**Table 5:** Barriers to and facilitators of responding to all of the residents’ care needs

In what circumstances the needs of residents are most commonly unmet	Macro‑, meso‑, and micro‑level factors influencing residential care facilities’ ability to respond to residents’ needs	
	Barriers	Facilitators
	**Macro-level (policy/ health system /contextual)**	
**Individual limitations** • Functional and sensory limitations • Physical health status • Lack of familial affection or love • Threat perception of autonomy • Complexity of needs	**Social and cultural factors** • Traditional social beliefs (teeth should be extracted and replaced with a removable prosthetic when aging) • Poor practical implementation of the integrated care policy	**Cohesive social environment** • Developing and maintaining a cohesive social environment • Family atmosphere
	**Public health systems & events** • Assessment of the problem of long-term care insurance system • COVID-19 pandemic and the associated limitations	**Care system** • Thorough and flexible care systems to meet the unique needs
	**Meso-level (organizations)**	
**Social and emotional** • Negative social experiences • Lack of social interaction • Inadequate social engagement	**Healthcare resources and services** • Inadequate healthcare resources and services • Further medical needs are difficult to meet • Difficulty in obtaining personal support	**Service optimisation and improvement** • Matching staffing to needs • Integration of needs and targeted services • Diversification of service content • Listening and responding to user needs and preferences • Communication and cooperation with each other in the service • Strengthening chronic care management
	**Human resources** • Shortage of human resources • Staff lack professionalisation, experience, awareness and training • Shortfall in the provision of specialised nursing and rehabilitation training	**Staff development and support** • Appropriate staff training and support
	**Institutional management** • Inadequate operation and management • Lack of appropriate reimbursement mechanism • Strict management systems	**Special population care** • Focused assessment and extra care for the highest risk groups
	**Physical environment and facilities** • Substandard buildings • Inadequate infrastructure and configuration • Tricky door-locking systems • Messy places and outdoor litter • Lack of introduction and identification of outdoor places	**Physical environment and accessibility** • Larger windows or patios • Electronic/automatic door openings and access to elevators for multiple floors • Access to balconies, patios, and conservatories
	**Micro-level (individual residents)**	
	**Trust and satisfaction** • Mistrust with the facility • Dissatisfaction with care	**Autonomy and participation** • Maintenance of the greatest possible perceived autonomy (ie, their participation) • Enhancing human care
		**Social support and connection** • Peer support • Strengthening social connections • Strengthening psychological support
		**Health education** • Health education that includes all stakeholders

#### Macro-level (policy/health system/contextual)

At the Macro-level, several structural and systemic barriers were identified that hinder the delivery of adequate care to older adults in residential care. These include socio-cultural factors such as traditional societal beliefs [83], and poor practical implementation of the integrated care policies [91]. In addition, challenges within public health systems & external events [67, 91] further exacerbate care limitations.

Traditional social and cultural beliefs can influence the oral health practices of older adults. The findings from Choufani et al. highlight a critical gap in oral healthcare for older adults [83]. In a survey of 526 older adults across 46 residential facilities in Lebanon, 84% had deposits on their dentures, indicating widespread, significant neglect of oral hygiene, which can lead to more severe dental and health issues over time. Moreover, although 57% of older people had unmet dental and restorative treatment needs, only 7% had sought treatment in the past year. This low treatment-seeking behaviour may be influenced by cultural norms that de-prioritise dental health, particularly among individuals accustomed to wearing dentures rather than maintaining natural teeth.

Macro-level barriers restricting the quality of long-term care services are the poor implementation of integrated care policies [91]. Yuan et al. found that most older adults lacked understanding of the integrated care policy and its specific content, making it difficult to connect the services they received with policy support [91]. Although integrated care policies have been incorporated into China’s national guiding documents, LTCFs often struggle to communicate these policies effectively to residents. This challenge arises largely from the absence of a coordination mechanism with government agencies and insufficient time and resources dedicated to policy dissemination [91, 92]. The discrepancy in policy execution has directly led to fragmented care services, creating a significant gap between policy intentions and actual delivery.

The influence of the public health system & external events on older person care services is substantial and cannot be overlooked. While the long-term care insurance (LTCI) system in China has played a role in safeguarding older people’s care rights and interests and alleviating the burden of family care, its evaluation mechanisms remain a source of controversy [91]. Yuan et al. found that most older adults reported loopholes in LTCI’s assessment processes for care needs and health status, which led to exploitation and detachment from their actual health conditions [91]. As one resident stated, “*When LTCI assessors arrived*,* I could have walked down the stairs but they made me use a wheelchair and pretend to be disability and dementia* [91].” Another resident also said, “*LTCI assessors only asked me two very simple questions*,* and after I answered*,* they just hurried to leave. I saw that they just wrote hypertension and diabetes in my evaluation results. However*,* my actual condition is much more serious than that* ” [91]. Moreover, public health emergencies such as the COVID-19 pandemic have further exposed the vulnerability of the residential care service system [67, 91]. During the pandemic, RCs generally adopted closed management to prevent and control infection, resulting in limited social interactions for the older people, obstructed medical services, and doubled pressure on caregivers [67, 91]. Especially in the early stages of the pandemic, problems such as shortages of protective materials, inadequate emergency plans, and poor information communication were exposed, further exacerbating the unmet psychological and social needs of older people.

The macro-level facilitators identified, namely the social environment [73, 91] and care systems [27] play a critical role in addressing the unmet needs of older adults in long-term care and residential care settings. Evidence suggests that when the broader social environment is cohesive and supportive, older adults are more likely to receive timely and appropriate care because staff are more engaged and responsive to residents’ concerns [73, 91]. In turn, this can lead to greater satisfaction and better health outcomes for residents, as unmet care needs are addressed more promptly and comprehensively [73]. In addition, Hancock et al. highlight the importance of a flexible and holistic care system, particularly in the light of the complex and diverse care needs of older adults [27]. The study emphasises that when care systems adopt a comprehensive approach that integrates health, psychological, and social services, they are better equipped to respond effectively to the care needs of older people.

#### Meso-level (organisations)

The review revealed several meso-level barriers that hinder the provision of adequate care for older adults in RC settings. These barriers included inadequate healthcare resources and services [76, 79, 84, 91], shortage of human resources [54, 79, 80, 82, 83, 85–87, 91], institutional management challenges [80, 81, 90] and limitations in the physical environment and facilities [67, 68, 80, 81]. The systemic issues impede the provision of comprehensive, responsive, and adequate care for older adults. Several studies have highlighted that a lack of healthcare infrastructure and services restricts access to essential care [76, 79, 91]. Inadequate healthcare resources mean that many facilities are ill-equipped to meet the complex needs of older adults. Moreover, the limited availability of diverse healthcare services, such as psychological counselling, music therapy, complementary therapies, or psychiatric care, further constrains the holistic and person centred care of older people [84]. When older people require specialised medical or personalised support, institutions often struggle to provide timely and effective responses, resulting in unmet care needs [91].

The shortage of human resources, particularly caregivers, is another critical barrier to timely and effective care [79, 80, 85, 87]. This issue is further exacerbated by insufficient professional training among staff, many of whom lack the skills needed to accurately assess, identify, and respond to the specific needs of older people [54, 79, 82, 83, 86]. For example, in Choufani et al.’s study of 46 residential facilities, only one had an on-site dental clinic, highlighting a substantial gap in access to oral health services [83]. A key contributing factor was limited awareness and training among nursing staff who serve as the primary caregivers for older residents [83]. Inadequate training has been shown to contribute to variability in care quality [82], and to undermine older adults’ trust in and satisfaction with, care services [77].

Institutional management barriers are equally significant. Some RC settings exhibit poor operational management and lack flexible, coordinated mechanisms for service coordination [80, 81, 90]. Lack of appropriate reimbursement mechanisms further compromises service continuity and quality [81]. Additionally, institutional policies, such as strict visitation restrictions and curfews, can undermine residents’ autonomy and negatively affect their well-being and participation in care [77]. These constraints contribute to unmet needs in social and vocational activities, exacerbating feelings of isolation and disconnection [67].

The physical environment and facilities of RC settings play a critical role in shaping older adults’ access to appropriate care and their overall well-being. Several studies have highlighted the significant variability in the quality of institutional buildings, with many facilities exhibiting inadequate infrastructure and poorly designed environmental features [67, 68, 80, 81]. Common issues reported in the review included complex door-locking systems, untidy outdoor environments, delayed waste removal, lack of clear signage and guidance aids [68, 80]. These deficiencies not only hinder daily convenience but also increase safety risks for older adults. Liljegren et al. emphasised that environmental design elements such as narrow windows, absence of balconies, patios, or conservatories, and lack of enclosed greenery in outdoor space design can negatively impact the comfort and psychological well-being of older people [68]. Furthermore, shared garden spaces while intended to foster community may inadvertently compromise privacy and reduce residents’ sense of belonging, diminishing their willingness to engage socially and emotionally with the environment [68].

In addition to barriers, meso-level facilitators were highlighted in the included studies. These include service optimisation and improvement [60, 64, 67, 82, 91], staff development and support [27, 79, 87], special population care [58, 70, 72], and enhancements to the physical environment and accessibility [68]. Collectively, these factors promote the personalisation, specialisation, and environmental adaptability of care services, serving as essential enablers for improving the quality of care for older people. Service optimisation is key to addressing the complex and evolving needs of older people. Studies have shown that aligning staffing to the care needs of older people [60], diversifying service offerings [67, 82], and integrating individual needs with targeted services [64] can significantly reduce unmet care needs. In addition, actively listening to and promptly responding to residents’ needs and preferences of service recipients [64, 67] have been shown to enhance care satisfaction of older people, foster a sense of respect and understanding. As one resident stated, “*They also listen and do things for us when we need something*” [67].

Staff development and support are also critical to enhancing service quality. Providing systematic training equips caregivers with the professional knowledge and practical skills to accurately identify and respond to the complex and personalised needs of older people, timely interventions, and personalised care planning [27, 87].

Finally, the physical environment and accessibility are foundational to enhancing the quality of life and care accessibility for older people. Studies have emphasised the importance of features such as larger windows or patios, electric/automatic doors, and multi-storey elevators, and access to open spaces, balconies, patios, and conservatories [68]. These environmental enhancements contribute to greater comfort and safety and opportunities for daily activities and social participation of older people [68].

#### Micro-Level (individual residents)

Barriers to care needs at the micro-level included trust and satisfaction problems [77]. Trust and satisfaction play a significant role in shaping the care experience of older people. David et al. found that most older people lack trust in RCs themselves and believe that the traditional health resources are inadequate in addressing their personal and specific mental health needs [77]. This lack of trust often leads to older people reluctance in expressing concerns or dissatisfaction, which in turn allows unmet needs to persist and further exacerbates feelings of isolation and neglect.

Facilitators of care needs at the micro-level were autonomy and participation [66, 91], social support and connection [67, 85], and health education [67]. Declining autonomy directly influences older adults’ life experience— As one resident in the Schweighart et al. study reported, “*loss of autonomy and independence as why he could no longer enjoy his life*”. However, he noted that “*he would be very willing to do so if he performs activities independently in a nursing home*” [67]. Related studies have also demonstrated that when older adults’ autonomy is fully respected, their willingness to communicate with caregivers increases, and their underlying needs can be more accurately identified and addressed [93, 94]. Furthermore, regular social activities and support groups help older adults establish deeper social connections, enhance their self-awareness, and encourage more proactive expression of personal needs and preferences, thereby improving well-being and reducing unmet care needs [67, 85].

## Discussion

The scoping review aimed to map and summarise the existing literature, identify unmet care needs among older people residing in RC settings, and explore the factors influencing the delivery of appropriate care. In contrast to the scoping review conducted by Kalánková et al. [35], which primarily focused on the concepts and terminology, methodological approaches, and ethical issues related to unmet care needs across various care settings, this scoping review explicitly restricted its scope to the residential care context. This review systematically synthesised factors influencing the provision of appropriate care at the individual, organisational, and policy levels. By focusing specifically on residential care settings and integrating evidence across macro, meso, and micro levels, this review extends existing knowledge by providing a context-specific and multilevel understanding of unmet care needs, thereby informing future research, policy development, and care practice in residential care environments.

This scoping review included 40 studies that described the broad and varied unmet care needs of older people living in RC settings, with the majority concentrated in four major domains: physical, psychological, social, and environmental. In terms of regional distribution, notable differences were observed in the types of unmet need across studies. Studies conducted in Europe and Asia frequently reported unmet social needs, such as companionship, social engagement, and emotional support, whereas North American studies frequently reported unmet physiological needs, such as mobility, self-care, psychological distress, and daytime activities. This distribution may reflect geographic or regional differences in research focus, shaped by contextual factors, measurement approaches, and prevailing research traditions. Although there are differences in the specific manifestations of unmet care needs among different regions, unmet physical and social needs have become a core challenge faced by older people worldwide, a finding that is consistent with a previous review by Kalánková et al. [35].

This scoping review highlights that the factors influencing access to appropriate care are multifaceted, spanning the macro, meso, and micro levels, and interacting in ways that form a complex interconnected system and reflect health disparities in geriatric care. At the macro-level, access to appropriate care in residential care facilities is influenced by sociocultural values and public health and long-term care policies. Sociocultural norms profoundly influence care priorities, service modalities, and the division of caregiving responsibilities between families and institutions. In China, although norms of familial obligation and filial piety have weakened over time, filial practices expressed through family-based caregiving continue to play a significant role, while institutional care is often regarded as a “last resort” [95]. This value orientation not only shapes the government’s policy priorities and resource allocation in long-term care but also influences older adults’ perceptions and expressions of their care needs and their acceptance of institutional services. Policy-related barriers further constrain the equity and continuity of care provision. Evidence indicates that weak implementation of integrated care policies and systemic deficiencies in long-term care insurance assessment frameworks, frequently result in fragmented service delivery, inefficient resource allocation, and unequal access to care across population groups [91]. In this context, ensuring service quality and accountability through institutional design has become a core governance issue facing the long-term care system.

Consumers of long-term care services often experience physical or cognitive impairments; they therefore may face significant barriers to switching providers when confronted with substandard care. Consequently, government-led regulation constitutes a critical mechanism for safeguarding the quality of care. By establishing clear quality standards, entry requirements, and ongoing regulatory oversight, governments can standardise facility operations and link quality performance to operating licenses or eligibility for public funding [96]. Internationally, many countries have strengthened long-term care regulation by developing standardised quality indicator systems. In the United States, the Improving Medicare Post-Acute Care Transformation Act was enacted in 2014, requiring all long-term care providers to report standardised data on service quality and resident outcomes [97]. Using the Minimum Data Set (MDS) 3.0 Resident Assessment Instrument (RAI) includes 24 core quality indicators covering domains such as functional status, clinical outcomes, and medication safety. These indicators are applied for regulatory oversight, quality improvement initiatives, and payment determinations [98]. Australia implemented the Aged Care Act 2024, which requires long-term care service providers to report 11 core quality indicators for each older person. These indicators focus on pressure injuries, falls, use of physical restraints, and unplanned weight loss, with the aim of enhancing service transparency and supporting continuous quality improvement [99]. In China, quality certification of care facilities is guided by the Basic Specification of Service Quality for Senior Care Organizations. In addition, local governments are encouraged to develop voluntary facility rating systems, building upon national standards while adapting to regional conditions [100]. However, ensuring service quality does not rely solely on regulatory mechanisms; Shankar et al. used a comprehensive probabilistic cost‑effectiveness analysis to compare robotic exoskeleton therapy with conventional physiotherapy, demonstrating how targeted interventions can improve outcomes while efficiently using resources [101].

Macro-level policies and social culture directly shape and constrain organizational capabilities at the meso-level. At the meso-level, organizational challenges prevalent in long-term care systems worldwide — such as insufficient healthcare resources, shortage of human resources, weak institutional management, and inadequate physical environment [54, 67, 68, 76, 79–87, 90, 91, 102] — are key drivers of inequities in care delivery, as facilities with fewer resources are less able to meet residents’ needs adequately [76, 79, 84, 91]. At the same time, the imbalance between service supply and demand has become an international challenge: on the one hand, the aging population is accelerating the demand for care; on the other hand, nursing work has long faced structural dilemmas such as low wages, high-intensity work, high turnover, and limited career development opportunities, making it difficult for long-term care institutions to stabilise and expand the labour supply [102, 103].

Inadequate staffing levels and high staff turnover not only directly reduce the amount of care time available and disrupt continuity of services, but also erode trust between care staff and residents, thereby undermining the relational care and its overall quality [104–107]. A national survey of 759 nursing homes in the United States highlighted that 87% of facilities experienced moderate to severe staffing shortages, and 73% reported concerns about potential closure due to workforce crises [108]. Data from Ohio further demonstrated workforce instability, with an average annual retention rate of 64% and an annual turnover rate of up to 55% [109]. To contain costs, some facilities have adopted subcontracting arrangements. This practice is associated with low wages, limited benefits, and insufficient training for care workers, which in turn accelerates the loss of experienced staff and contributes to declining care quality [110]. High turnover is often accompanied by staff reductions, excessive workloads, and frequent role changes, leaving care workers with insufficient time to develop an in-depth understanding of residents’ individual needs or to respond to those needs adequately [111]. The prevalence of subcontracting is closely linked to inadequate public funding for long-term care. Existing evidence indicates that for-profit long-term care facilities generally exhibit lower staffing levels and poorer care quality than non-profit or publicly operated facilities [112, 113]. This is because for-profit institutions often control costs and maximise profits by maintaining minimal staffing levels, suppressing wages, and replacing highly skilled workers with less experienced personnel [114, 115]. In response, proposed policy-level solutions include prohibiting or restricting subcontracting practices and requiring for-profit operators to re-enter provincial collective bargaining frameworks to improve labour conditions [110]. Alternatively, increasing government subsidies may enable facilities to remain financially viable without compromising staffing adequacy or care quality [110].

Adequate staffing is a fundamental prerequisite for the delivery of care and the maintenance of care quality. Evidence demonstrated that higher staffing levels and more optimal skill mixes are associated with better care outcomes [116]. Currently, many countries have enacted legislation to clarify staffing standards and access to qualification systems to improve service quality. United States federal regulations require all nursing homes to provide a minimum of 3.48 h of direct care per day and mandate the continuous presence of a registered nurse 24 h a day, seven days a week, to ensure the continuity of professional nursing care [117]. Australia has introduced comparable regulatory measures, stipulating that each residential care facility must ensure that at least one registered nurse is always on-site and maintain an appropriate ratio of nursing assistants [118]. Australia requires all nurses to have qualifications in gerontology, hold valid professional licenses, demonstrate relevant skills or experience, and provide criminal background clearance [118].

As the older population continues to grow, the health conditions encountered in care settings are becoming increasingly complex, placing higher demands on the professional competencies of care staff. Beyond meeting minimum staffing requirements, enhancing workforce education and training has therefore become a critical priority. Maas et al. suggested that, while ensuring basic staffing, registered nurses should be provided with systematic leadership training, and geriatric care training to improve the overall care competency of the team [119]. At the organisational level, long-term care facilities should establish routine and ongoing in-service training mechanisms to continuously update care workers’ knowledge and competencies, enabling them to respond effectively to evolving care demands [120]. At the governmental level, greater investment is required to expand funding for education and training programmes, thereby increasing the pool of potential workers with appropriate professional skills [121].

Organisational capacity and institutional arrangements at the meso level, particularly staffing levels, workforce skills, resource availability and service coordination mechanisms, shape care delivery at the micro level, directly determining whether individual needs of older adults can be effectively identified and addressed. At the micro-level, insufficient trust in care staff or care institutions [77] continuously undermine older adults’ capacity to articulate their needs and to access appropriate care. In contrast, autonomy, participation, and social support are key enabling factors. Person-centred care is widely recognised as an effective approach to strengthening and respecting older adults’ autonomy [122, 123], and as a key strategy for tackling healthcare inequities [124]. In the United States, federal regulations explicitly require nursing homes to support residents in directing their own care, ensuring that care plans are not developed unilaterally by institutions but instead fully incorporate residents’ preferences and choices [125]. These regulations mandate that interdisciplinary care plan meetings be held at least quarterly, with participation by residents, family members, and direct care staff. Ireland similarly mandates protecting the right of older persons to participate in care decisions and ensuring services are appropriately tailored to individual health conditions and social needs [126]. In addition, the findings from Jolande et al. revealed that facilities had established formal mechanisms to guarantee residents’ right to participate, including the establishment of resident committees and working committees [123]. The facilities also specified procedures that allow older adults to access electronic care plans and regular care plan review meetings. However, whether individual needs at the micro level can be adequately met largely depends on organisational capacity at the meso level and on policy/ health system, and contextual factors at the macro level. In other words, whether older adults receive adequate care in RC settings ultimately reflects the availability of macro-level policies and their translation into meso-level practices for the individual resident.

In summary, appropriate care in residential care facilities is the result of the dynamic interplay among macro-level policy and cultural factors, meso-level organisational effectiveness, and micro-level individual agency. These three levels do not operate in isolation but are interconnected and mutually reinforcing. Ineffective policy implementation and macro-level socio-cultural orientations exacerbate resource shortages and hinder access to personal support at the meso level. Conversely, gaps in organisational implementation at the meso level hinder the effective feedback of policy implementation at the macro level. Unmet care needs of older adults at the micro level ultimately manifest as the cumulative outcome of issues originating from the other two levels. Therefore, only through multi-level collaborative strategies increasing government investment and strengthening regulatory systems (macro level), optimising human resource allocation and professional training (meso level), and enhancing older adults’ self-participation (micro level)—can a person-centred and sustainable long-term care system be established, thereby effectively improving the well-being of older adults.

### Strengths and limitations

The strength of this scoping review is that it expands understanding of the unmet care needs of older people in RC settings and systematically summarises the multi-level factors that influence the delivery of appropriate care, using a micro-meta-macro framework. However, as with all evidence synthesis, the current scoping also has some limitations. First, in line with the JBI scoping review methodology, no formal methodological quality appraisal was conducted for the included studies. While this approach enables a comprehensive mapping of the existing evidence, methodological heterogeneity across studies was not systematically assessed, which may affect the robustness and interpretation of the findings. Second, the inclusion studies published only in selected languages introduces linguistic and geographical limitations, meaning that unmet care needs in other cultural or regional contexts may not be fully represented.

## Conclusions

This review found that older adults living in RC settings have multiple unmet care needs, with regional variations in the types and distribution of these needs, which are jointly influenced by multi-level factors at the micro, meso, and macro levels. Based on these findings, several recommendations are proposed. First, the Camberwell Assessment of Need for the Elderly (CANE) should be introduced as a standardised screening instrument to systematically identify multidimensional unmet needs among older people in RC settings. Second, stratified and individualised intervention strategies should be developed to reflect regional variations. Third, cross-sectoral collaboration mechanisms should be established to integrate resources from the health, social security, and civil affairs, enabling more precise responses to older adults’ care needs.

## Supplementary information

Supplementary Material 1.

Supplementary Material 2.

## Data Availability

The datasets supporting the conclusions of this article are included within the article (and its additional file).
