# Supplementary material for: Unmet care needs among older people in residential care: a scoping review
Source: BMC Geriatr. 2026 May 12;26:912. doi: 10.1186/s12877-026-07636-y (PMC13339487; doi:10.1186/s12877-026-07636-y)
Supplement: Supplementary file 1 — Supplementary Material 1. [file 12877_2026_7636_MOESM1_ESM.docx]

**Additional file 1**

Search of **EMBASE** **(Elsevier)** conducted on 2nd March 2024.

| Search | Query | Records Retrieved |
| --- | --- | --- |
| #1 | 'needs assessment'/exp | 27,811 |
| #2 | 'unmet care need*':ab,ti OR 'unmet need*':ab,ti OR 'needs assessment*':ab,ti OR 'assessment of need*':ab,ti OR 'health need assess*':ab,ti OR 'assessment of health need*':ab,ti OR 'healthcare needs assess*':ab,ti OR 'assessment of healthcare need*':ab,ti OR 'health care needs assess*':ab,ti OR 'assessment of health care need*':ab,ti OR 'health services needs and demand*':ab,ti OR 'health services accessibility*':ab,ti | 52,019 |
| #3 | ((need* NEAR/2 assess*):ab,ti) OR ((need* NEAR/2 unmet*):ab,ti) OR ((need* NEAR/2 disparit*):ab,ti) OR ((need* NEAR/2 healthcare):ab,ti) OR ((need* NEAR/2 'health care'):ab,ti) OR ((demand* NEAR/2 assess*):ab,ti) OR ((demand* NEAR/2 unmet*):ab,ti) OR ((demand* NEAR/2 disparit*):ab,ti) OR ((demand* NEAR/2 healthcare):ab,ti) OR ((demand* NEAR/2 'health care'):ab,ti) | 136,913 |
| #4 | #1 OR #2 OR #3 | 155,062 |
| #5 | 'geriatrics'/exp OR 'aged'/exp OR 'aging'/exp OR 'longevity'/exp OR 'retirement'/exp | 4,180,264 |
| #6 | older:ab,ti OR 'older people':ab,ti OR 'older adult*':ab,ti OR 'older person*':ab,ti OR elder:ab,ti OR elderly:ab,ti OR senior:ab,ti OR geriatric*:ab,ti OR gerontol*:ab,ti OR aged:ab,ti OR aging:ab,ti OR longevity:ab,ti OR retire*:ab,ti OR octogenarian*:ab,ti OR centenarian*:ab,ti | 2,281,781 |
| #7 | #5 OR #6 | 5,408,828 |
| #8 | 'residential care'/exp OR 'residential home'/exp OR 'long term care'/exp OR 'nursing home'/exp OR 'assisted living facility'/exp | 2,490,337 |
| #9 | 'residential care*':ab,ti OR 'residential home*':ab,ti OR 'residential care home*':ab,ti OR 'residential care facilit*':ab,ti OR 'residential care institution*':ab,ti OR 'residential facilit*':ab,ti OR 'long-term care':ab,ti OR 'long term care':ab,ti OR 'long-term care facilit*':ab,ti OR 'long term stay home*':ab,ti OR 'nursing home*':ab,ti OR 'retirement communit*':ab,ti OR 'retirement facilit*':ab,ti OR 'retirement home*':ab,ti OR 'assisted living':ab,ti OR 'assisted living facilit*':ab,ti OR 'assisted care facilit*':ab,ti OR 'old folks home*':ab,ti OR 'old peoples home*':ab,ti OR 'convalescent home*':ab,ti OR 'rest home*':ab,ti | 84,939 |
| #10 | #8 OR #9 | 2,512,517 |
| #11 | #4 AND #7 AND #10 | 6,148 |

Search of **CINAHL (EBSCOhost)** conducted on 2nd March 2024.

| Search | Query | Records Retrieved |
| --- | --- | --- |
| S1 | (MH “Needs Assessment”) OR (MH “Health Services Needs and Demand”) OR (MH “Health Services Accessibility”) | 152,570 |
| S2 | TI (“unmet care need*” OR “unmet need*” OR “Needs Assessment*” OR “assessment of need*” OR “health need assess*” OR “assessment of health need*” OR “healthcare needs assess*” OR “assessment of healthcare need*” OR “health care needs assess*” OR “assessment of health care need*” OR “Health Services Needs and Demand*” OR “Health Services Accessibilit*”) OR AB (“unmet care need*” OR “unmet need*” OR “Needs Assessment*” OR “assessment of need*” OR “health need assess*” OR “assessment of health need*” OR “healthcare needs assess*” OR “assessment of healthcare need*” OR “health care needs assess*” OR “assessment of health care need*” OR “Health Services Needs and Demand*” OR “Health Services Accessibilit*”) | 15,212 |
| S3 | TI ((need* OR demand*) N2 (assess* OR unmet* OR disparit* OR healthcare OR “health care”)) OR AB ((need* OR demand*) N2 (assess* OR unmet* OR disparit* OR healthcare OR “health care”)) | 52,033 |
| S4 | S1 OR S2 OR S3 | 191,369 |
| S5 | (MH Geriatrics) OR (MH Aged) OR (MH Aging) OR (MH Longevity) OR (MH Retirement) OR (MH Octogenarians) OR (MH Centenarians) | 983,523 |
| S6 | TI (older OR “older people” OR “older adult*” OR “older person*” OR elder OR elderly OR senior OR Geriatric* OR gerontol* OR Aged OR Aging OR Longevity OR Retire* OR Octogenarian* OR Centenarian*) OR AB (older OR “older people” OR “older adult*” OR “older person*” OR elder OR elderly OR senior OR Geriatric* OR gerontol* OR Aged OR Aging OR Longevity OR Retire* OR Octogenarian* OR Centenarian*) | 604,887 |
| S7 | S5 OR S6 | 1,297,619 |
| S8 | (MH “Residential Care”) OR (MH “Residential Facilities”) OR (MH “Long Term Care”) OR (MH “Nursing Homes”) | 59,730 |
| S9 | TI (“Residential Care*” OR “residential home*” OR “residential care home*” OR “residential care facilit*” OR “residential care institution*” OR “Residential facilit*” OR “long-term care” OR “Long Term Care” OR “long-term care facilit*” OR “long term stay home*” OR “Nursing Home*” OR “retirement communit*” OR “retirement facilit*” OR “retirement home*” OR “Assisted Living” OR “assisted living facilit*” OR “assisted care facilit*” OR “old folks home*” OR “old folks' home*” OR “old people's home*” OR “convalescent home*” OR “rest home*”) OR AB (“Residential Care*” OR “residential home*” OR “residential care home*” OR “residential care facilit*” OR “residential care institution*” OR “Residential facilit*” OR “long-term care” OR “Long Term Care” OR “long-term care facilit*” OR “long term stay home*” OR “Nursing Home*” OR “retirement communit*” OR “retirement facilit*” OR “retirement home*” OR “Assisted Living” OR “assisted living facilit*” OR “assisted care facilit*” OR “old folks home*” OR “old folks' home*” OR “old people's home*” OR “convalescent home*” OR “rest home*”) | 49,051 |
| S10 | S8 OR S9 | 80,920 |
| S11 | S4 AND S7 AND S10 | 2,872 |

Search of **PSYCINFO (EBSCOhost)** conducted on 2nd March 2024.

| Search | Query | Records Retrieved |
| --- | --- | --- |
| S1 | (DE “Needs Assessment”) | 10,040 |
| S2 | TI (“unmet care need*” OR “unmet need*” OR “Needs Assessment*” OR “assessment of need*” OR “health need assess*” OR “assessment of health need*” OR “healthcare needs assess*” OR “assessment of healthcare need*” OR “health care needs assess*” OR “assessment of health care need*” OR “health services needs and demand*” OR “health services accessibility”) OR AB (“unmet care need*” OR “unmet need*” OR “Needs Assessment*” OR “assessment of need*” OR “health need assess*” OR “assessment of health need*” OR “healthcare needs assess*” OR “assessment of healthcare need*” OR “health care needs assess*” OR “assessment of health care need*” OR “health services needs and demand*” OR “health services accessibility”) | 10,660 |
| S3 | TI ((need* OR demand*) N2 (assess* OR unmet* OR disparit* OR healthcare OR “health care”)) OR AB ((need* OR demand*) N2 (assess* OR unmet* OR disparit* OR healthcare OR “health care”)) | 34,102 |
| S4 | S1 OR S2 OR S3 | 40,589 |
| S5 | (DE Geriatrics) OR (DE Aging) OR (DE Retirement) | 109,538 |
| S6 | TI (older OR “older people” OR “older adult*” OR “older person*” OR elder OR elderly OR senior OR Geriatric* OR gerontol* OR aged OR Aging OR longevity OR Retire* OR octogenarian* OR centenarian*) OR AB (older OR “older people” OR “older adult*” OR “older person*” OR elder OR elderly OR senior OR Geriatric* OR gerontol* OR aged OR Aging OR longevity OR Retire* OR octogenarian* OR centenarian*) | 551,239 |
| S7 | S5 OR S6 | 563,763 |
| S8 | (DE “Residential Care Institutions”) OR (DE “Long Term Care”) OR (DE “Nursing Homes”) OR (DE “Retirement Communities”) OR (DE “Assisted Living”) | 28,820 |
| S9 | TI (“residential care*” OR “residential home*” OR “residential care home*” OR “residential care facilit*” OR “Residential Care Institution*” OR “residential facilit*” OR “long-term care” OR “Long Term Care” OR “long-term care facilit*” OR “long term stay home*” OR “Nursing Home*” OR “Retirement Communit*” OR “retirement facilit*” OR “retirement home*” OR “Assisted Living” OR “assisted living facilit*” OR “assisted care facilit*” OR “old folks home*” OR “old folks' home*” OR “old people's home*” OR “convalescent home*” OR “rest home*”) OR AB (“residential care*” OR “residential home*” OR “residential care home*” OR “residential care facilit*” OR “Residential Care Institution*” OR “residential facilit*” OR “long-term care” OR “Long Term Care” OR “long-term care facilit*” OR “long term stay home*” OR “Nursing Home*” OR “Retirement Communit*” OR “retirement facilit*” OR “retirement home*” OR “Assisted Living” OR “assisted living facilit*” OR “assisted care facilit*” OR “old folks home*” OR “old folks' home*” OR “old people's home*” OR “convalescent home*” OR “rest home*”) | 26,729 |
| S10 | S8 OR S9 | 38,492 |
| S11 | S4 AND S7 AND S10 | 608 |

Search of **MEDLINE (EBSCOhost)** conducted on 2nd March 2024.

| Search | Query | Records Retrieved |
| --- | --- | --- |
| S1 | (MH “Needs Assessment”) OR (MH “Health Services Needs and Demand”) OR (MH “Health Services Accessibility”) | 163,317 |
| S2 | TI (“unmet care need*” OR “unmet need*” OR “Needs Assessment*” OR “assessment of need*” OR “health need assess*” OR “assessment of health need*” OR “healthcare needs assess*” OR “assessment of healthcare need*” OR “health care needs assess*” OR “assessment of health care need*” OR “Health Services Needs and Demand*” OR “Health Services Accessibilit*”) OR AB (“unmet care need*” OR “unmet need*” OR “Needs Assessment*” OR “assessment of need*” OR “health need assess*” OR “assessment of health need*” OR “healthcare needs assess*” OR “assessment of healthcare need*” OR “health care needs assess*” OR “assessment of health care need*” OR “Health Services Needs and Demand*” OR “Health Services Accessibilit*”) | 31,873 |
| S3 | TI ((need* OR demand*) N2 (assess* OR unmet* OR disparit* OR healthcare OR “health care”)) OR AB ((need* OR demand*) N2 (assess* OR unmet* OR disparit* OR healthcare OR “health care”)) | 113,964 |
| S4 | S1 OR S2 OR S3 | 261,444 |
| S5 | (MH Geriatrics) OR (MH Aged) OR (MH Aging) OR (MH Longevity) OR (MH Retirement) OR (MH Octogenarians) OR (MH Centenarians) | 3,628,416 |
| S6 | TI (older OR “older people” OR “older adult*” OR “older person*” OR elder OR elderly OR senior OR Geriatric* OR gerontol* OR Aged OR Aging OR Longevity OR Retire* OR Octogenarian* OR Centenarian*) OR AB (older OR “older people” OR “older adult*” OR “older person*” OR elder OR elderly OR senior OR Geriatric* OR gerontol* OR Aged OR Aging OR Longevity OR Retire* OR Octogenarian* OR Centenarian*) | 1,654,355 |
| S7 | S5 OR S6 | 4,546,385 |
| S8 | (MH “Residential Facilities”) OR (MH “Long-Term Care”) OR (MH “Nursing Homes”) OR (MH “Assisted Living Facilities”) | 69,935 |
| S9 | TI (“residential care*” OR “residential home*” OR “residential care home*” OR “residential care facilit*” OR “residential care institution*” OR “Residential Facilit*” OR “Long-Term Care” OR “long term care” OR “long-term care facilit*” OR “long term stay home*” OR “Nursing Home*” OR “retirement communit*” OR “retirement facilit*” OR “retirement home*” OR “assisted living” OR “Assisted Living Facilit*” OR “assisted care facilit*” OR “old folks home*” OR “old folks' home*” OR “old people's home*” OR “convalescent home*” OR “rest home*”) OR AB (“residential care*” OR “residential home*” OR “residential care home*” OR “residential care facilit*” OR “residential care institution*” OR “Residential Facilit*” OR “Long-Term Care” OR “long term care” OR “long-term care facilit*” OR “long term stay home*” OR “Nursing Home*” OR “retirement communit*” OR “retirement facilit*” OR “retirement home*” OR “assisted living” OR “Assisted Living Facilit*” OR “assisted care facilit*” OR “old folks home*” OR “old folks' home*” OR “old people's home*” OR “convalescent home*” OR “rest home*”) | 66,327 |
| S10 | S8 OR S9 | 98,784 |
| S11 | S4 AND S7 AND S10 | 3,855 |

Search of **Web of Science (Core Collection)** conducted on 2nd March 2024.

| Search | Query | Records Retrieved |
| --- | --- | --- |
| #1 | TS=(“unmet care need*” OR “unmet need*” OR “needs assessment*” OR “assessment of need*” OR “health need assess*” OR “assessment of health need*” OR “healthcare needs assess*” OR “assessment of healthcare need*” OR “health care needs assess*” OR “assessment of health care need*” OR “health services needs and demand*” OR “health services accessibilit*”) | 39,018 |
| #2 | TS=(need* NEAR/2 assess* OR need* NEAR/2 unmet* OR need* NEAR/2 disparit* OR need* NEAR/2 healthcare OR need* NEAR/2 'health care' OR demand* NEAR/2 assess* OR demand* NEAR/2 unmet*OR demand* NEAR/2 disparit* OR demand* NEAR/2 healthcare OR demand* NEAR/2 'health care') | 128,639 |
| #3 | #1 OR #2 | 130,794 |
| #4 | TS=(older OR “older people” OR “older adult*” OR “older person*” OR elder OR elderly OR senior OR geriatric* OR gerontol* OR aged OR aging OR longevity OR retire* OR octogenarian* OR centenarian*) | 5,703,348 |
| #5 | TS=(“residential care*” OR “residential home*” OR “residential care home*” OR “residential care facilit*” OR “residential care institution*” OR “residential facilit*” OR “long-term care” OR “long term care” OR “long-term care facilit*” OR “long term stay home*” OR “nursing home*” OR “retirement communit*” OR “retirement facilit*” OR “retirement home*” OR “assisted living” OR “assisted living facilit*” OR “assisted care facilit*” OR “old folks home*” OR “old folks' home*” OR “old people's home*” OR “convalescent home*” OR “rest home*”) | 83,337 |
| #6 | #3 AND #4 AND #5 | 1,255 |

Search of **CNKI** conducted on 2nd March 2024.

| Search | Query | Records Retrieved |
| --- | --- | --- |
| S1 | (TI='未满足的护理需求' OR TI='未满足的需求' OR TI='未满足需求' OR TI='未满足照护需求' OR TI='需求评估' OR TI='健康需求评估' OR TI='医疗保健需求评估' OR TI='保健需求评估' OR TI='保健服务需要和需求' OR TI='保健服务可及性' OR TI='需求 /NEAR 2 评估' OR TI='需求 /NEAR 2 未满足' OR TI='需求 /NEAR 2 差距' OR TI='需求 /NEAR 2 医疗保健' OR TI='需要 /NEAR 2 评估' OR TI='需要 /NEAR 2 未满足' OR TI='需要 /NEAR 2 差距' OR TI='需要 /NEAR 2 医疗保健') OR (AB='未满足的护理需求' OR AB='未满足的需求' OR AB='未满足需求' OR AB='未满足照护需求' OR AB='需求评估' OR AB='健康需求评估' OR AB='医疗保健需求评估' OR AB='保健需求评估' OR AB='保健服务需要和需求' OR AB='保健服务可及性' OR AB='需求 /NEAR 2 评估' OR AB='需求 /NEAR 2 未满足' OR AB='需求 /NEAR 2 差距' OR AB='需求 /NEAR 2 医疗保健' OR AB='需要 /NEAR 2 评估' OR AB='需要 /NEAR 2 未满足' OR AB='需要 /NEAR 2 差距' OR AB='需要 /NEAR 2 医疗保健') | 1,476,000 |
| S2 | (TI='老人' OR TI='老年人' OR TI='老年' OR TI='年老' OR TI='年长' OR TI='老年学' OR TI='老者' OR TI='老龄化' OR TI='长寿' OR TI='退休' OR TI='八旬老人' OR TI='百岁老人') OR (AB='老人' OR AB='老年人' OR AB='老年' OR AB='年老' OR AB='年长' OR AB='老年学' OR AB='老者' OR AB='老龄化' OR AB='长寿' OR AB='退休' OR AB='八旬老人' OR AB='百岁老人') | 20,681,000 |
| S3 | (TI='养老院' OR TI='养护院' OR TI='护养院' OR TI='疗养院' OR TI='护理院' OR TI='福利院' OR TI='敬老院' OR TI='老年公寓' OR TI='老人院' OR TI='长期照护' OR TI='长期护理' OR TI='长期照护设施' OR TI='长期照护机构' OR TI='辅助生活' OR TI='老人之家' OR TI='托老所' OR TI='康复之家' OR TI='退休社区') OR (AB='养老院' OR AB='养护院' OR AB='护养院' OR AB='疗养院' OR AB='护理院' OR AB='福利院' OR AB='敬老院' OR AB='老年公寓' OR AB='老人院' OR AB='长期照护' OR AB='长期护理' OR AB='长期照护设施' OR AB='长期照护机构' OR AB='辅助生活' OR AB='老人之家' OR AB='托老所' OR AB='康复之家' OR AB='退休社区') | 1,544,000 |
| S4 | S1 AND S2 AND S3 | 27 |

Search of **WANFANG Data** conducted on 2nd March 2024.

| Search | Query | Records Retrieved |
| --- | --- | --- |
| S1 | (题名或关键词:(“未满足的护理需求” OR “未满足的需求” OR “未满足需求” OR “未满足照护需求” OR “需求评估” OR “健康需求评估” OR “医疗保健需求评估” OR “保健需求评估” OR “保健服务需要和需求” OR “保健服务可及性” OR ((“需求” OR “需要”) AND (“评估” OR “未满足” OR “差距” OR “医疗保健”)))) | 8,263 |
| S2 | (主题:(“未满足的护理需求” OR “未满足的需求” OR “未满足需求” OR “未满足照护需求” OR “需求评估” OR “健康需求评估” OR “医疗保健需求评估” OR “保健需求评估” OR “保健服务需要和需求” OR “保健服务可及性” OR ((“需求” OR “需要”) AND (“评估” OR “未满足” OR “差距” OR “医疗保健”)))) | 381,007 |
| S3 | (题名或关键词:(“老人” OR “老年人” OR “老年” OR “高龄” OR “年老” OR “年长” OR “老年学” OR “老者” OR “老龄化” OR “长寿” OR “退休” OR “八旬老人” OR “百岁老人”)) | 1,026,979 |
| S4 | (主题:(“老人” OR “老年人” OR “老年” OR “高龄” OR “年老” OR “年长” OR “老年学” OR “老者” OR “老龄化” OR “长寿” OR “退休” OR “八旬老人” OR “百岁老人”)) | 1,419,204 |
| S5 | (题名或关键词:(“养老院” OR “养护院” OR “护养院” OR “疗养院” OR “护理院” OR “福利院” OR “敬老院” OR “老年公寓” OR “老人院” OR “长期照护” OR “长期护理” OR “长期照护设施” OR “长期照护机构” OR “辅助生活” OR “老人之家” OR “托老所” OR “ 康复之家” OR “退休社区”)) | 29,673 |
| S6 | (主题:(“养老院” OR “养护院” OR “护养院” OR “疗养院” OR “护理院” OR “福利院” OR “敬老院” OR “老年公寓” OR “老人院” OR “长期照护” OR “长期护理” OR “长期照护设施” OR “长期照护机构” OR “辅助生活” OR “老人之家” OR “托老所” OR “ 康复之家” OR “退休社区”)) | 42,631 |
| S7 | S1 AND S3 AND S5 | 46 |
| S8 | S2 AND S4 AND S6 | 1,077 |
| S9 | S7 OR S8 | 1,123 |

Search of **ProQuest Dissertations and Theses Global** conducted on 2nd March 2024.

| Search | Query | Records Retrieved |
| --- | --- | --- |
| S1 | AB,TI (“unmet care need*” OR “unmet need*” OR “needs assessment*” OR “assessment of need*” OR “health need assess*” OR “assessment of health need*” OR “healthcare needs assess*” OR “assessment of healthcare need*” OR “health care needs assess*” OR “assessment of health care need*” OR “health services needs and demand*” OR “health services accessibilit*” OR ((need* OR demand*) N2 (assess* OR unmet* OR disparit* OR healthcare OR “health care”))) | 59,771 |
| S2 | AB,TI (older OR “older people” OR “older adult*” OR “older person*” OR elder OR elderly OR senior OR geriatric* OR gerontol* OR aged OR aging OR longevity OR retire* OR octogenarian* OR centenarian*) | 186,988 |
| S3 | AB,TI (“residential care*” OR “residential home*” OR “residential care home*” OR “residential care facilit*” OR “residential care institution*” OR “residential facilit*” OR “long-term care” OR “long term care” OR “long-term care facilit*” OR “long term stay home*” OR “nursing home*” OR “retirement communit*” OR “retirement facilit*” OR “retirement home*” OR “assisted living” OR “assisted living facilit*” OR “assisted care facilit*” OR “old folks home*” OR “old folks' home*” OR “old people's home*” OR “convalescent home*” OR “rest home*”) | 7,283 |
| S4 | S1 AND S2 AND S3 | 1,441 |
