# Supplementary material for: Unmet care needs among older people in residential care: a scoping review
Source: BMC Geriatr. 2026 May 12;26:912. doi: 10.1186/s12877-026-07636-y (PMC13339487; doi:10.1186/s12877-026-07636-y)
Supplement: Supplementary file 2 — Supplementary Material 2. [file 12877_2026_7636_MOESM2_ESM.docx]

**Additional file 2** Scoping review study characteristics

| **Authors & year & Country** | **Aims** | **Study design** | **Data collection method** | **Sample size** | **Data analysis** | **Main findings** |
| --- | --- | --- | --- | --- | --- | --- |
| **Quantitative** | | | | | | |
| Hawkins et al. (1998) [69]  Canada | To describe the oral health status and treatment needs of nursing home and independently living residents aged 85 years and older in North York, Canada. | Cross sectional observation | Questionnaire and visual screening examination | n=1313 residents from nursing home n=65 residents from independent | Descriptive statistics | High levels of unmet need were identified in subjects from both nursing home and independent residences. Among nursing home residents, about 45% of dentate subjects required tooth extraction, and 56% required prosthodontic treatment. Among independently living residents, about 27% of dentate subjects required tooth extraction, over 60% needed restorative treatment. Higher prevalence of both untreated decay and unmet treatment needs was associated with lower utilization of dental care for dentate subjects. |
| Yee et al. (1999) [70]  USA | To understand how resident choice, getting needed care, and a sense of community were promoted or hindered. | Cross sectional survey | In-person interview | n=396 residents at 20 assisted living (AL) settings | Descriptive statistics | AL residents were older, less likely to use Medicaid, and more likely to live with others (during or before AL residence) than older people in the other populations identified as at risk for needing long-term care. |
| Martin et al. (2002) [54]  UK | To compare needs (met and unmet) and levels of dependency in residential care (RC) and nursing care (NC) settings. | Cross sectional survey | CANE, Clifton Assessment Procedure for the Elderly-Behaviour Rating Scale (CAPE-BRS), and Mini-Mental State Examination  (MMSE) | n=34 residents living in specialist elderly mentally ill nursing care (NC) settings (a hospital- based continuing-care ward and a health-funded private nursing home) n=40 residents living in residential care (RC) settings | Descriptive statistics | A high number of needs were found in both RC and NC settings. The average number of 14.2 in total needs and 2.6 unmet needs for RC residents, they had more unmet needs for both assistances with daytime activities, and psychological distress (p<0.05). The average number of 14.4 in total needs and 2.5 unmet needs for NC residents, they had more unmet needs for memory.  The results show that NC residents had a greater level of overall dependency than the RC group as well as greater levels of cognitive impairment. |
| Hancock et al. (2006) [27]  UK | To identify the unmet needs of people with dementia in care and the characteristics associated with high levels of needs. | Cross sectional survey | CANE, CAPE-BRS, Barthel Scale of Activities of Daily Living, Challenging Behaviour Scale (CBS), Cornell Scale for Depression in Dementia, Rating Anxiety In Dementia (RAID), Clinical Dementia Rating scale (CDR), and MMSE | n=238 residents with dementia | Descriptive statistics | Residents with dementia had a mean of 4.4 unmet needs. Sensory or physical disability (including mobility problems and incontinence) needs, mental health needs, and social needs, such as company and daytime activities, were often unmet. Younger residents (p < 0.05) and people who had resided in the home for a shorter period of time (p < 0.05) had more unmet needs. Unmet needs were associated with psychological problems, such as anxiety and depression (p < 0.05), but not with severity of dementia or level of dependency. |
| Kiely et al. (2010) [72]  USA | To identify characteristics of nursing home (NH) residents with advanced dementia and their healthcare proxy (HCP) associated with hospice referral and to examine the association between hospice use and the treatment of pain and dyspnea and unmet needs during the last 7 days of life. | Prospective cohort study | Telephone interviews  Toolkit After-Death Bereaved Family Member Interview | n=323 NH residents with advanced dementia and their healthcare proxies. | Descriptive statistics | 22.3% of NH residents were referred to hospice. Factors independently associated with hospice referral were non-white race (AOR=2.55, 95% CI=1.36–4.76), eating problems (AOR=4.21, 95% CI=1.99–8.90), HCP perception that resident had less than 6 months to live (AOR=3.83, 95% CI=1.29–11.37), and better HCP mental health on the SF-12 (AOR=1.04, 95% CI=1.01–1.06). Treatment of pain with scheduled opioids was significantly more likely when residents were in hospice (48.3% vs 19.4%; AOR=3.16, 95% CI=1.57–6.36). A total of 68.3% of residents with hospice care had an unmet need in at least one domain, compared with 81.3% who did not receive hospice services. |
| Van der Ploeg et al. (2013) [31]  Netherlands | To compare the number and type of unmet needs of people with and without dementia in residential care in the Netherlands using the Camberwell Assessment of Needs for the Elderly (CANE) interview (24 topics). | Cross sectional survey | Cognitive Performance Scale, Camberwell Assessment of Needs in Elderly (CANE), and Groningen Activity Restriction Scale | n=93 residents without dementia  n=58 residents with dementia who completed the CANE themselves  n=36 residents with dementia for whom a proxy completed the CANE | Descriptive analyses | People diagnosed with dementia reported more total needs and unmet needs (an average of 7.11 in total and 0.39 unmet needs) than people without dementia (an average of 6.46 in total and 0.14 unmet needs), and within the dementia group proxies reported more needs (an average of 10.33 in total and 0.83 unmet needs) than residents themselves.  With the non-dementia group as reference group showed that the three groups significantly differed in the number of needs reported for accommodation, money, medication management, benefits, incontinence, memory problems, inadvertent self-harm, company and daytime activities (p<0.05). |
| Mitchell (2013) [73]  USA | To investigate the existence and prevalence of specific unmet functional care needs in assisted living homes and identify their predictors. | Cross sectional survey | Self-reported unmet care needs were designed after items in the National Health Interview Survey of 1994, used by the US National Center for Health Statistics. | n=201 residents | Descriptive statistics and regression models | 28% of residents reported at least 1 activities of daily living (ADL) unmet need and 27% reported at least 1 instrumental activities of daily living (IADL) unmet need.  Predictors of unmet ADL needs were ADL dependency (z=8.01, p<0.01) and cohesion subscale (z=19.16, p<0.001).  Predictors of unmet IADL needs were IADL dependency (z=4.27, p<0.05) and cohesion subscale (z=7.15, p<0.01). |
| Roszmann et al. (2014) [57]  Poland | Aimed at the characterization of the met and unmet needs of the elderly living in long-term care facilities and also to learn about the living conditions of older people living in institutions, focusing on their various needs. | Cross sectional survey | CANE | n=98 residents with dementia | Descriptive statistics | The average number of needs identified met and unmet for all older adults was 20.4, and 17.8. The most commonly unmet needs concerned ‘accommodation’ (96.9%) – people were not satisfied with the place where they lived, ‘memory’ (95.9%) – clear deficits in recalling new information, one more was ‘food’ (72.4%) – dissatisfaction with the type of help and problems with swallowing were reported. |
| Mazurek et al. (2015) [59]  Poland | To analyse the complex needs of residents of nursing homes over 75 years old in different Polish cities from different perspectives and to explore the unmet need associations of health-related factors. | Cross sectional survey | CANE, MMSE, and the Geriatric Depression Scale (GDS). | n=300 residents over 75 years old n=300 rater n=300 staff | Descriptive statistics | Comparison of needs examined from three different perspectives (the nursing home resident, the rater and nursing home staff) showed that nursing home residents reported significantly fewer problems (7.84±3.20) than nursing home staff (9.71±3.01) and rater (9.49±2.20), but significantly more unmet needs (1.32±1.47) than staff (0.51±0.92). Moreover, the more severe the depression and the worse the cognitive functions were, the more unmet needs were reported by nursing home residents. |
| Szczepańska-Gieracha et al. (2015) [60]  Poland | To analyse the phenomenon of disability in the oldest age group and the needs related with it, depending on the place of residence of elderly people. | Cross sectional survey | CANE, GDS, MMSE, Barthel Index (BI), The Timed ‘‘Up & Go’’, and socio-demographic and clinical questionnaire. | n=100 residents from nursing home n=70 residents from family home environment | Descriptive statistics | From the respondent's point of view there were no significant differences in the assessment of needs that were met, unmet, and in total number of needs, whereas from the researcher's point of view greater number of both met needs (P < 0.001), unmet needs (P = 0.001) as well as total needs (p < 0.001) were recorded in a NH. In the outpatient (family home environment) group, as opposed to NH group, no differences were recorded between opinions of the respondent and researcher in both the met and unmet needs. Among patients who came from a NH environment, the number of met needs positively correlated with the functional status of older people (the better the functional status of the patient as well as his cognitive state, the more both met and unmet needs). Among patients who came from family home environment the number of met needs negatively correlated with the functional status (the greater the degree of disability, the lesser the met needs). |
| Nikmat and Almashoor (2015) [78]  Malaysia | To identify the needs of people with cognitive impairment living in nursing homes and factors associated with higher level of needs. | Cross sectional survey | Short MMSE, CANE, BI, Friendship Scale, and GDS | n=110 residents with cognitive impairment aged ≥60 | Descriptive statistics | Respondents had a mean of 2.81 for unmet needs. The most frequent unmet needs reported were social needs (i.e.: intimate relationships, company, caring for another), sensory or physical disability (i.e.: daytime activity, eyesight/hearing, mobility) and environmental needs (including food, medication used, information, abuse/neglect and money). Unmet needs were significantly associated with depression, social isolation, and cognitive impairment. |
| Wieczorowska-Tobis et al. (2016) [61]  Poland | To evaluate the CANE in assessing the needs of elderly individuals living in LTCI in Poland. | Cross sectional survey | MMSE, BI, GDS, and CANE | n=173 residents (aged ≥75 years) | Descriptive statistics | The number of met needs assessed by the staff was higher (9.3 ± 3.0) than in the users’ opinions (7.8 ± 3.2, p < 0.0001), whereas the number of unmet needs was lower (0.6 ± 0.9) than in the users’ opinions (1.3 ± 1.4, p < 0.001). The average agreement rate between users and staff perception of needs with CANE was 86.2%. There was lowest agreement between staff and users in relation to company (65.3%), memory (75.7%), eyesight/hearing/communication (70.5%) and psychological distress (70.5%). |
| Ferreira et al. (2016) [62]  Portugal | To describe the needs of an institutionalised sample of residents and to analyse its relationship with demographic and clinical characteristics. | Cross sectional survey | CANE, MMSE, Geriatric Depression Scale 15 items (GDS-15), IAFAI, and NPI | n=175 residents living in nursing home | Descriptive statistics | 170 (97.1%) out of 175 residents presented one or more unmet needs.  Statistically significant negative correlations (p<0.05) were found between MMSE score and met, unmet and global needs. Significant correlations (p<0.05) between depressive symptoms and unmet, between behavioural and psychological symptoms (BPSD) and unmet, and between functional impairment and unmet. |
| Wang (2017) [79]  China | To explore the needs and needs fulfilment of older people and to analyse the influence factors. | Cross sectional survey | Activities of daily living Sale, and the needs of the elderly questionnaire in nursing home | n=367 residents | Descriptive statistics | The mean score of total needs of older people in nursing home was (31.31 ± 9.521), was 48.92% that was in middle level. In aspect of physiological needs, the main unfulfilled needs were rehabilitation training with the help of medical staff, dental examination and oral care, sleep problems, and bathing assistance. In terms of safety needs, the unfulfilled need of reimbursement about nursing treatment, care and other services. In terms of love and belonging needs, the main unfulfilled needs were chatting and demand of the staffs’ company. In terms of self-esteem needs, the main unfulfilled needs were the needs of social activities. In terms of self-fulfilment needs, the main unfulfilled needs were taking part in recreational activities, and cleaning. Self-care ability, the conditions about chronic diseases, the census register, economic sources, the charge standard, education background were the main influencing factors of older people needs (p<0.05). |
| Tobis et al. (2018) [63]  Poland | To investigate the patterns of needs in older individuals living in long-term care institutions (LTCIs) using CANE questionnaire. | Cross sectional survey | MMSE, BI, GDS, and CANE | n=306 residents (age: ≥75 years) with MMSE score of at least 10 points | Descriptive statistics | Residents had 10.4±3.2 met needs and 0.8±1.2 unmet needs. The most commonly unmet needs included company (15.9%), psychological distress (14.0%), intimate relationship (11.4%), eyesight/hearing/communication (11.4%), and daytime activities (11.0%). The study shows that the OR of having a large number of unmet needs did not depend on BI nor on GDS. With the OR for people with an MMSE score of 10–19 (i.e., moderate dementia symptoms) being more than four times higher than for people with an MMSE score of 24–30 (i.e., without dementia symptoms). |
| Ferreira et al. (2020) [64]  Portugal | To explore neuropsychiatric symptoms’ (NPS) risk factors in a sample of nursing home residents. | Cross sectional survey | Neuropsychiatric Inventory (NPI), MMSE, the Adults and Older Adults Functional Assessment Inventory (IAFAI), and CANE | n=140 residents over 65 years | Descriptive statistics | 50.4% residents presented at least one NPS. NPI-10 showed significant correlations with cognition (p = 0.042), functional impairment (p = 0.043), unmet needs (p = 0.004) and nervous system-acting drugs (p = 0.002). An average of 3.91 ± 2.11 needs were unmet. Those unmet needs including daytime activities, sensory disability, psychological distress and company. |
| Choufani et al. (2020) [83]  Lebanon | Assess the oral health of Lebanese people aged 65 years and over living in residential facilities, and identify factors associated with poor oral status. | Cross sectional observation | Questionnaire and clinical oral examination | n=526 residents | Descriptive statistics | 55.9% of the older adults were edentates, 41.4% used partial and/or complete dentures, and the minority used dentures with good hygiene. 57% of the older adults presented with an unmet need for a dental prosthesis. Among the edentulous sample, more than 40% did not use any denture and around 10% used only one denture when in fact they were in need for both. Oral health status was significantly related to age, smoking, daily tooth brushing, and autonomy (p < 0.05). |
| Rivera et al. (2020) [75]  USA | To examine the factors associated with increased depressive symptoms among a pool of older adults, with a focus on change in reported needs after starting long-term services and supports (LTSS). | Longitudinal cohort | Registry data | n=352 residents | Descriptive statistics and regression models | Depressive symptoms were present among 40% of the LTSS recipients at enrolment and 3 months. At baseline, 29% of LTSS recipients reported a need for supportive equipment, 30% for transportation, and 23% for social activities. After 3 months, an average of 12% of LTSS recipients’ needs were met, 13% of LTSS recipients’ needs persisted, and 11% of LTSS recipients reported new needs. At three months depressive symptoms were higher for those who reported persistent unmet needs compared with those who reported no needs at all, controlling for functional status and LTSS type. |
| Duan et al. (2020) [76]  USA | To identify the prevalence of unmet preferences and unimportant preferences and examine their associations with resident and facility-level characteristics. | Longitudinal cohort | Registry data | n=25,668 residents (51,859 assessments) | Descriptive statistics and regression models | Across all years for both daily routine preferences and activity preferences, 3.3% to 5.1% of residents reported that at least 1 or more preferences was important but unmet, and 10.0% to 16.6% reported that 4 or more out of the 8 preferences were unimportant. Residents with higher depressive symptoms, and poorer physical and sensory function were more likely to report unmet preferences (p<0.05). Residents with poorer physical and sensory function and living in rural facilities and facilities having fewer activity staff hours per resident day were more likely to report unimportant preferences (p<0.05). |
| Tobis et al. (2021) [65]  Poland | Applied a cluster approach to investigate whether distinct cognitive, functional and psychological profiles exist, which can distinguish the needs of residents. | Cross sectional survey | MMSE, BI, GDS, and CANE | n=83, Cluster 1 (C1) (MMSE=23.7 ± 4.4, BI = 85.8 ± 14.4, GDS= 3.3 ± 2.0).  n=87, Cluster 2 (C2) (MMSE=21.0 ± 4.0, BI=79.8 ± 15.1, GDS = 8.9 ± 2.1)  n=72, Cluster 3 (C3) (MMSE=18.3 ± 3.1, BI=30.6 ± 18.8, GDS =7.6 ± 2.3) | Descriptive statistics | The number of met needs was higher in C2 than in C1 (10.0 ± 3.2 vs 8.2 ± 2.7, p < 0.001), and in C3 (12.1 ± 3.1) than in both C1 and C2 (p < 0.001). The number of unmet needs was higher in C3 than in C1 (1.2 ± 1.5 vs 0.7 ± 1.0, p = 0.015).  In the area Daytime activities, C3 had more unmet needs in comparison with both C1 and C2 (p = 0.023 and p = 0.003, respectively). There were also differences in the area’s Eyesight/hearing/communication (C2 vs. C3, p = 0.022) and psychological distress (C1 vs. C3, p = 0.032). |
| Huang et al. (2022) [87]  China | To understand the characteristics of unmet needs of older adults residing in nursing homes in China, and to probe into the contributing factors. | Cross sectional survey | CANE, the Short Portable Mental Status Questionnaire, Demographic and health status questionnaire, Modified Barthel Index, the Numerical Rating Scale for pain assessment, and the GDS‑15 | n=2063 residents | Descriptive statistics and regression models | 122 older adults having more than 3 unmet needs (high unmet need category) versus 1922 older ones having ≤ 3 unmet needs (low unmet need category). The unmet needs of older adults in nursing homes mainly fell into social domains. gender, religion, educational background, marital status, living condition before admission, room type, incomes, staffing, number of diseases, pain, Barthel Index, and depression contribute to unmet needs of older adults in long-term care facilities. |
| **Qualitative** | | | | | | |
| Wang et al. (2019) [81]  China | To understand the long term care needs of the elderly from the aged care institutions in Chongqing. | Phenomenology | Semi—structured in depth interviews | n=12 residents | Content analysis | Long-term care needs can be categorised as care for physical functioning, psychosocial support, maintenance of social functioning, and creation of an environment. However, some of these needs are not being met. |
| Song et al. (2020) [84]  China | To explore residents’ (unmet) care needs in pain and related functional limitations. | Descriptive qualitative study | Semi-structured open-ended interviews | N=21 residents living in residential care facilities | Descriptive statistics and thematic analysis | Residents described significant unmet care needs related to pain and functional limitations. About half reported moderate or severe pain and the other half reported mild pain. Of the 21 residents, 19 reported functional limitations caused by pain, and only one of them reported receiving assistance with ADL from staff. |
| Chammem et al. (2021) [66]  France | To better understand the experiences, perceptions, coping mechanisms, and needs of older people living in Martinique who had to modify their living environment because of a decline of autonomy. | Qualitative | Semi-structured interviews | n=9 residents living at home n=13 residents living in foster care family n=12 residents living in nursing home | Content analysis | The study found differences between the three groups regarding familial relationship, fear of death, acceptance of change, and unmet needs. Family and social relationships appeared to be more important determinants of older adults’ perception of changes in living environment than was health status. People living in foster care families particularly expressed the unmet needs of occupational activities, lack of warm relationships, lack of outings, the authority of the hosts. |
| Schweighart et al. (2022) [67]  Germany | To examine the needs of nursing home residents with depressive symptoms and the communication of those needs. | Explorative cross-sectional qualitative study | Semi-structured interviews | n=11 residents with depressive symptoms | Content analysis | NH residents with depressive symptoms have diverse and complex needs (care provision, dying and death, health, hobbies and preferences, independence and autonomy, move out and relocation, no needs, occupation and daily structure, psychological and emotional needs, reminiscence, service and facilities, and social needs), many of which are unmet. Barriers such as health impairments, absence of social contacts or negative social experiences prevented the fulfilment of needs.  In addition, lack of confidants, missing or non-functioning communication tools, impatience and perceived lack of understanding on the part of caregivers, and residents’ insecurities limit communication of needs. |
| Lee et al. (2023) [89]  China | To examine Chinese cultural impediments to nursing home residents signing advance directives. | Descriptive qualitative | Semi-structured interview | n=18 residents | Content analysis | This study found that the impediments to signing an advance directive are mainly related to Chinese culture, specifically the death taboo, a family-centric worldview, and the influence of Buddhism and Taoism. Several older adults indicated that they were unable or unwilling to sign because their individual needs were not met, including individualised educational discussions and consideration of personal choice. |
| Yuan et al. (2024) [91]  China | To explore the perceptions and experiences of integrated care among older residents living in long-term care facilities. | Descriptive qualitative study | Semi-structured in-depth interviews | N=18 older adults from 5 long-term care institutions | Content analysis | Five themes, including (1) Policy level: positive attitude and negative feelings. (2) Physiological level: satisfied basic daily life, primary medical services accessibility and chronic care management enhancement needed. (3) Psychological level: need for psychological support and need for self-perception recognition. (4) Relationship level: enhancement of humanistic care and need for a family atmosphere. (5) Social level: interpersonal communication constrained by the times and inadequate social engagement.  Older adults expressed dissatisfaction with advanced medical services, specialised chronic disease management, psychological support, humanistic care, and social participation. |
| Liljegren et al. (2024) [68]  Sweden | To explore the needs and wishes of older adults concerning their perceived need for contact with outdoor environments at residential care facilities (RCFs) and what implications it has for theory and practice. | Descriptive qualitative study | Semi-structured interviews conducted in the form of walking interviews | N=12 older adults from three Swedish RCFs | Qualitative content analysis | Older adults used and expressed needs and wishes for contact with the outdoors. The first category, Outdoor environments as part of everyday life. The second category, Getting outdoors in practice.  RC densification, tricky door-locking systems, transitions between zones, etc. hinder their opportunities for outdoor stays.  Older people have difficulty in accessing personal support. Older people who are dependent on carers, relatives or acquaintances to move in and between zones did not get the same opportunity for outdoor stays as independent older adults. |
| **Mixed method** | | | | | | |
| Pretty (2014) [58]  UK | Presents a contextual approach to the assessment of dental health needs based on a life course approach with vulnerability integrated within the assessment of need. | Mixed methods | Questionnaire, and interview | n=424 residents living in residential homes n=244 residents living in nursing homes n=188 residents living in dual (residential and nursing) homes | Descriptive statistics and content analysis | The unmet need for dental care is high amongst residents in residential and nursing homes in Trafford. Several interviewees pointed out that they needed domiciliary dental care due to dementia or mental health issues.  Most residents rely on carers to help them find dentists, but many homes lack dentist resources to contact, making it difficult for residents to get dental services when needed. This study shown that more than (61% (n=500) of people, needed a dental check-up or professional care but unmet. |
| Lu (2018) [80]  China | To organise and carry out planned social work service activities to improve the life confidence of the welfare homes and the elderly, meet their spiritual needs, and enrich their spiritual life in their later years. | Mixed method | Questionnaire, observation and interview | n=13 residents | Descriptive analysis and inductive analysis | There are problems such as lack of emotional support, pessimistic thoughts, monotony of entertainment, and less social participation in the five-guarantee elderly’s spiritual life of the welfare institutions. The author intervenes in the mental life problems of the five older people of the welfare homes by means of individual cases, groups, and community work methods. For example: carry out group and community activities to establish interpersonal relationships among older people. The research shown that the intervention can improve the negative lives of the five-guarantee elderly in the welfare institutions. State and increase their well-being. |
| Ma (2019) [82]  China | To reduce the loneliness of the elder living in nursing home. | Mixed method | Questionnaire, interview and observation | N=34 residents | Descriptive statistics; Content analysis. | 76.5% of older people feel lonely, but their needs are not being met. The reason for the loneliness of self-care older people is that they have more leisure time but nothing to do, while semi self-care older people had obstacles in action and negative psychology. Interventions of exercise and singing activities for the self-care older people, and manual group activities for the semi-self-care older people. This study found that after intervention, the average score of loneliness decreased 7.07 from 38.96. |
| Duan (2021) [85]  China | To study the current situation and needs of the "five guarantees" in the mental comfort of the elderly in T nursing homes | Mixed method | Observation, interview and questionnaire | n=23 residents | Descriptive analysis and inductive analysis | The study found that T Nursing Home has problems such as low bed occupancy rate, extremely imperfect facilities and equipment, few staff and low level of professionalism, and lack of medical staff. The older people in the nursing home are in interpersonal communication, cultural entertainment, emotional comfort of need urgent but not met. |
| Chen (2022) [88]  China | Understanding the spiritual needs of the older people in Lanzhou apartment for the aged. | Mixed method | Interview and observation | n=10 staff members, nurses  n=19 older people | Descriptive analysis and inductive analysis | Due to limited interpersonal communication, weak social support network, and fewer entertainment and cultural activities, older people in the apartment for the aged are difficult to meet their spiritual needs in social communication, entertainment and culture, and generally have low mood, negative attitude and loneliness. The author identified 10 older people as the service objects of the group for 8 activities intervention. After group intervention, loneliness of group members generally decreased. |
| Zhu (2022) [86]  China | To discusses the spiritual comfort service for older people in rural areas. | Mixed method | Questionnaire, observation and unstructured interview | n=19 residents | Descriptive analysis and inductive analysis | The lack of spiritual life of older people is manifested in the lack of security, low self-identity, and unfulfilled cultural recreation and social communication. The reasons for this are related to the poor health of older people themselves and their family situation without children. |
| Huang (2023) [90]  China | To understand the adaptation problems of the five guarantee older people in nursing home and to identify the current status of their adaptation and its causes. | Mixed method | Semi-structured interviews and participatory observation | n=20 residents (Five guarantees refer to the elderly, weak, lonely and disabled residents in rural areas who lack family support, lack of the ability to work and the source of income) | Descriptive analysis and inductive analysis | The five guarantee older people had adaptation problems in daily life, psychological and emotional levels, and interpersonal communication levels. Specifically in daily life, the diet in nursing homes was relatively simple, which cannot meet their needs for variety and taste. The study found that the main influencing factors for older people in the nursing home to have difficulty adapting are the lack of daily care services, strict management systems in the nursing home, unclear self-awareness of the elderly with five guarantees, and a decline in their interpersonal skills after moving into the nursing home, as well as weak social support networks. |
| David et al. (2023) [77]  USA | To investigate mental health needs and barriers to seeking mental health support in Medicaid-funded Assisted Living Facility (M-ALF). | Mixed method | Questionnaires and semi structured interviews, and observational field notes/memos | n=13 residents living in Medicaid-funded Assisted Living Facility | Descriptive statistics; Conventional content analysis. | Residents reported barriers to mental health access. 10 of 13 respondents demonstrated mental health need, but none of the respondents were currently receiving professional mental health support. Barriers to mental health support including dissatisfaction with M-ALF care, perceived threats to autonomy, desire for autonomy that leads to diminished care seeking. |
| **Randomised controlled trial (RCT)** | | | | | | |
| Orrell et al. (2007) [55]  UK | To reduce unmet needs in older people with dementia in residential care compared to a ‘care as usual’ control group. | Randomised controlled trial | CANE, Quality of Life in Alzheimers Disease, MMSE, CDR, CAPE-BRS, Barthel Scale of Activities of Daily Living, CBS, Cornell Scale for Depression in Dementia, and RAID | 238 people aged 60+ with dementia living in 24 residential homes from three areas  n=118 people with intervention (1 hour per week liaison input per home to deliver a personalised intervention package over a 20 weeks) n=120 people with control condition (care as usual) | Descriptive statistics | At follow-up the mean number of unmet needs for people with dementia in the intervention homes reduced by 3.1 (standard deviation, SD 2.0), compared to a reduction of 0.7 (standard deviation 2.9) unmet needs in the control group. |
| Orrell et al. (2008) [56]  UK | To compare the ratings of needs of older people with dementia living in care homes, as assessed by the older person themselves, a family caregiver, and the staff of the care home. | Randomised controlled trial | CANE, MMSE, CDR, CAPE-BRS, Barthel Scale of Activities of Daily Living, Cornell Scale for Depression In Dementia, and RAID | n=149 residents (users) n=81 family carers n=238 staff | Descriptive statistics | Of the 24 possible needs in CANE, the mean number of needs identified by staff (n=238) was 14.99, comprising 1.41 unmet needs. Family caregivers (n = 81) identified a mean of 14.49 needs, comprising 2.06 unmet needs. The mean number of needs identified by users (n = 149) was 10.21comprising 1.69 unmet needs. Users reported relatively higher unmet needs for psychological distress, company and information, daytime activities, and eyesight/hearing problems. |
| Cohen-Mansfield et al. (2015) [74]  USA | To describe the unmet needs of persons with dementia exhibiting behavioural problems. | Randomised controlled trial (RCT) | Direct observation  MMSE, the Pain Assessment in Elderly Persons, the Agitated Behaviours Mapping Instrument, Lawton's Modified Behaviour Stream, the Type of Unmet Need Assessment | n=89 residents with dementia RAs (research assistants) group (who observed residents and also implemented nonpharmacologic interventions) NAs (nursing assistants) group (front-line caregivers) | Descriptive statistics | The agreement rate between RAs and NAs averaged 86% for all types of needs, and kappa coefficient averaged 0.25. RAs reported an average of 2.9 unmet needs per older adult (range 0–7, S.D.=1.3) and NAs reported an average of 2.6 unmet needs per older adult (range 0–6, S.D.=1.4). RAs׳ designation of the unmet need of pain was associated with significantly better cognitive function as tapped by the MMSE, significantly higher levels of negative affect at baseline, and higher scores on the PAINE (p<0.05). |
| **Quasi-experimental** | | | | | | |
| Kane et al. (2002) [71]  USA | To compare the characteristics of a sample of EverCare nursing home residents with two control groups: one composed of other residents in the same homes and another made up of residents in matched nursing homes. To compare levels of unmet need, satisfaction with medical care, and the use of advance directives. | Quasi-experimental | In-person surveys and telephone surveys | n= 454 EverCare patients (experimental groups) (more intensive primary care is provided by using salaried NPs) n=407 EverCare Control (from among the residents in the same nursing homes participating in the EverCare program but opting not to join) n= 440 Other controls (usual medical care) (from nursing homes in the same geographic area that were not participating in EverCare) | Descriptive statistics | The EverCare sample had more dementia and less ADL disability. EverCare residents with more unmet need than controls in many instances. EverCare residents reported a significantly higher level of unmet need in transferring (EverCare vs EverCare Control, p=0.032). But family members in the EverCare sample expressed greater satisfaction with several aspects of the medical care they received than did controls. There was no difference in experience with advance directives between EverCare and control groups. |

Key: CANE= Camberwell Assessment of Needs in Elderly; RC= residential care; NC= nursing care; MMSE= Mini-Mental State Examination; NH= nursing home; HCP= healthcare proxy; AOR= adjusted odds ratio; CI= Confidence Interval; NPS= neuropsychiatric symptoms; GDS= Geriatric Depression Scale; NPI= Neuropsychiatric Inventory; GDS-15= Geriatric Depression Scale 15 items; IAFAI= Adults and Older Adults Functional Assessment Inventory; BI= Barthel Index; LTCIs=long-term care institutions; ADL= activities of daily living; IADL= instrumental activities of daily living; LTSS= long-term services and supports; AL= assisted living; CAPE-BRS= Clifton Assessment Procedures for Elderly-Behaviour Rating Scale; CBS= Challenging Behaviour Scale; RAID= Rating Anxiety In Dementia; CDR= Clinical Dementia Rating scale; M-ALF= Medicaid-funded Assisted Living Facility
